# Tailoring Two-Dimensional Matter Using Strong Light–Matter Interactions

**DOI:** 10.1021/acs.nanolett.2c04467

**Published:** 2023-03-06

**Authors:** Ye-Jin Kim, Yangjin Lee, WonJae Choi, Myeongjin Jang, Won-Woo Park, Kwanpyo Kim, Q-Han Park, Oh-Hoon Kwon

**Affiliations:** † Department of Chemistry, College of Natural Sciences, Ulsan National Institute of Science and Technology (UNIST), 50 UNIST-gil, Ulsan 44919, Republic of Korea; ‡ Center for Soft and Living Matter, Institute for Basic Science (IBS), 50 UNIST-gil, Ulsan 44919, Republic of Korea; § Department of Physics, Yonsei University, 50 Yonsei-ro, Seoul 03722, Republic of Korea; ∥ Center for Nanomedicine, IBS, 50 Yonsei-ro, Seoul 03722, Republic of Korea; ⊥ Department of Physics, Korea University, 145 Anam-ro, Seoul 02841, Republic of Korea

**Keywords:** black phosphorus, light-coupled *in situ* transmission electron microscopy, light−matter interactions, modulation instability, nanopatterning, nanoribbon, two-dimensional matter, wide-field optical lithography

## Abstract

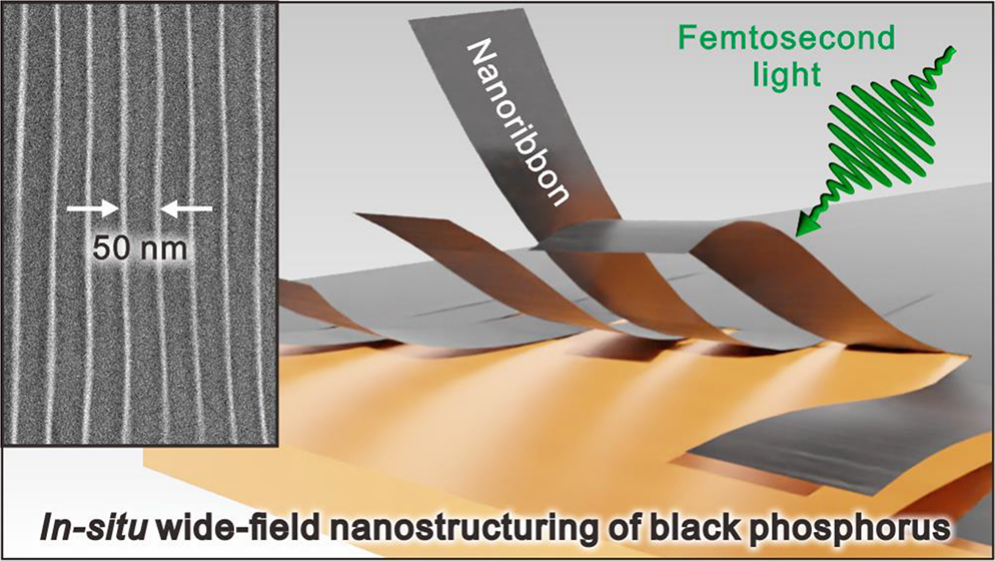

The shaping of matter into desired nanometric structures with on-demand functionalities can enhance the miniaturization of devices in nanotechnology. Herein, strong light–matter interaction was used as an optical lithographic tool to tailor two-dimensional (2D) matter into nanoscale architectures. We transformed 2D black phosphorus (BP) into ultrafine, well-defined, beyond-diffraction-limit nanostructures of ten times smaller size and a hundred times smaller spacing than the incident, femtosecond-pulsed light wavelength. Consequently, nanoribbons and nanocubes/cuboids scaling tens of nanometers were formed by the structured ablation along the extremely confined periodic light fields originating from modulation instability, the tailoring process of which was visualized in real time via light-coupled *in situ* transmission electron microscopy. The current findings on the controllable nanoscale shaping of BP will enable exotic physical phenomena and further advance the optical lithographic techniques for 2D materials.

Following an era of graphene, the rational design and alteration of architectures based on two-dimensional (2D) materials is at the forefront of research interest for the development of new physical and chemical properties beyond those of bulk counterparts.^1−4^ Presently, the further reduction of their dimensionality and creation of ultrafine nanostructures are gaining significance for engineering electronic structures and enhancing quantum confinement,^5−7^ harnessing them as new opportunities in scalable nanophotonic and optoelectronic devices.^8−10^

In context, a variety of methods have been employed for shaping 2D matter at nanoscales. Although bottom-up techniques involving chemical vapor deposition and site-selective growth have been demonstrated to achieve precise control and scalability,^11,12^ the reproducible fabrication for complex device structures and device integration are still unmet. In contrast, photolithography,^13^ electron beam lithography (EBL),^14^ and ion beam lithography^15^ are typical top-down techniques, but they entail cumbersome steps of etching or liftoff. Although scanning optical patterning is resist-free, high-throughput, and versatile,^16^ the optical diffraction limit of approximately half the light wavelength restricts its application in the areas where sub-100 nm precision is required.

In this study, we demonstrate that wide-field illumination by intense, ultrashort-pulsed light can “tailor” 2D matter into 1D and even quasi-0D nanostructures, with a spatial precision far beyond the optical diffraction limit—achieving up to 2 orders of magnitude smaller than the wavelength of the incident light approaching the dimension of quantum effects (Figure 1a). The formation of laser-induced periodic surface patterns on bulk matter by utilizing strong light–matter interaction has remained a long-standing subject with mechanisms therein regarded as disputable.^17−19^ As compared with bulk matter, the periodic structures developed in 2D matter under pulsed light were more elaborate and controllable with improved confined light–matter interactions.

**Figure 1:**
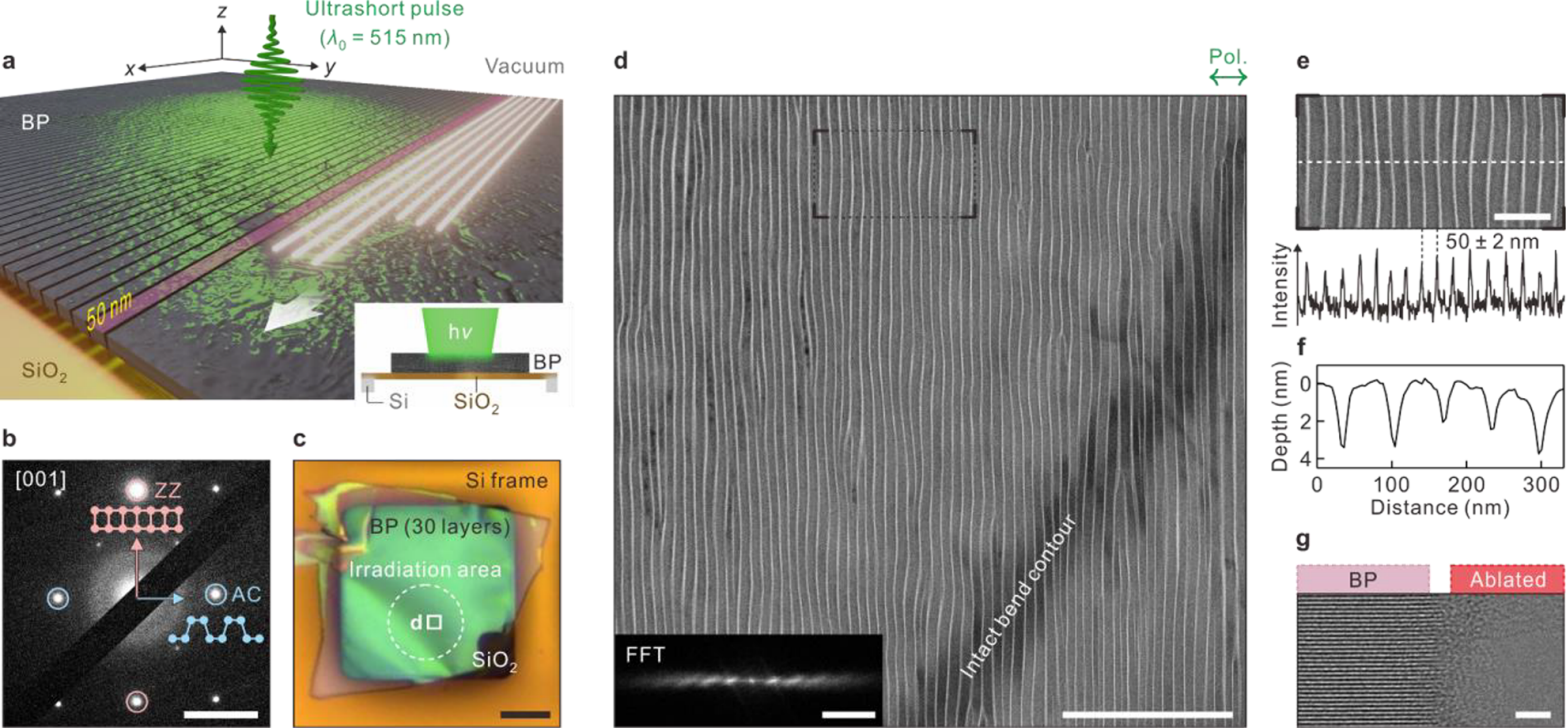
Laser-induced *in situ* nanosculpting BP. (a) Schematic illustration of wide-field nanosculpting BP with femtosecond-pulsed light. Incident light induces self-organized optical fields in BP, resulting in highly regular nanostructures over micrometer area by structured periodic ablation with a width of approximately 50 nm. Inset: side view of experimental layout. (b) Selected-area diffraction pattern of BP flake in panel c; scale bar: 5 1/nm. Anisotropic crystal structure containing AC (010) and ZZ (100) lattices. (c) Optical micrograph of 30-layered single-crystalline BP flake (sample 1). Dotted circle indicates irradiated area. Scale bar: 20 μm. (d) Low-magnification bright-field TEM image of the BP nanoribbon array at marked position (square) in panel c. Orientation (grating vector) of the array is parallel to light polarization (denoted above panel). Crystalline axes (AC and ZZ) are denoted. Scale bar: 1 μm. Inset: fast-Fourier transform of array; scale bar: 50 μm^–1^. (e) Enlarged view of marked region in panel d. Intensity profile across array (dashed line). Typical width of nanoribbon is 50 nm; scale bar: 200 nm. (f) AFM profile of the BP nanoribbon array (sample 2). (g) High-resolution bright-field TEM image of nanoribbon edge (sample 3); scale bar: 2 nm.

The current demonstration was conducted on black phosphorus (BP), conventionally exfoliated from the bulk, as a single-elemental 2D system with strong in-plane anisotropy in optical, vibrational, electronic, thermal, and mechanical aspects along the two orthogonal armchair (AC) vs zigzag (ZZ) axes (Figure 1b). Upon reducing its dimension to 1D with synthetic approaches or modeling,^20,21^ the resulting BP nanoribbons delivered extraordinary physical performances.^22^ In contrast to the bottom–up synthetic growth of nanostructures along the preferential crystallographic axes, we were able to tailor BP nanoribbons in any orientation between the AC and ZZ axes that exhibited intermediate properties to those of the two axes. We show that the strong nonlinear response of BP toward intense pulsed irradiation induced modulation instability (MI) of electromagnetic waves within it, which accompanied dense, self-organized optical fields across the thin layers and periodically ablated a collection of phosphorus (P) atoms along the footprint of the fields. The nanoscale tailoring process was filmed in real space and time via *in situ* light-coupled transmission electron microscopy (TEM)^23^ which provided rich information on the shaping mechanism.

Single-crystalline multilayer BP (typically 20–30 layers) was mechanically exfoliated into micrometer-sized flakes and transferred onto Si-support TEM grids covered with dielectric membrane of 8 nm thick SiO_2_ (Figures 1a–d). The sample information is detailed in Figure S1 and Table S1. The *in situ* light-coupled TEM allowed the illumination of the samples with focused femtosecond (fs)-pulsed light (full width at half-maximum, FWHM = 30 μm) with tunable parameters of wavelength (λ_0_), polarization, fluence (*F*), and number of pulses (*N*).

At a surface normal incidence (3–4°), the BP flakes at room temperature under vacuum environment were irradiated using multiple 515 nm pulses (*N*: 10^4^–10^5^) of 550 fs duration with linear polarization. Consequently, the flakes underwent periodically structured ablation to form highly regular linear nanostructures over a micrometer-scale area (>10 × 10 μm^2^), as depicted in Figure 1d (see Figure S2 for low-magnification TEM images). Generally, the fluence was set within the narrow range of 90 ≤ *F* ≤ 100 mJ/cm^2^. For low fluence, the energy confined along the periodic field was insufficient for inducing local ablation, whereas the entire mass under the illumination area was ablated for excessively high fluence. The repetition rate of the pulsed illumination was set to 1 kHz to avoid heat accumulation in the steady state. The array of “nanoribbons” with its orientation (grating vector) parallel to the polarization of the incident light could be tailored along any axes between 0° (AC) and 90° (ZZ) (Figure S3). Further experimental details are presented in the Methods section.

The real-space bright-field (BF) TEM image of a well-organized array of BP nanoribbons from sample 1 formed at *F* = 90 mJ/cm^2^ is illustrated in Figure 1d. The real-time formation of the nanoribbons is visualized in Movie S1. The nanoribbons featured an aspect ratio higher than 100 with a typical width and length of 50 nm (Figure 1e) and >5 μm, respectively. Based on the dislocation features such as bending, bifurcation, and junction along the ablated lines (Figure S4), the spatial uniformity of nanoribbons was apparently affected by the surface topography, but microscale morphology was intact as the bend contour was continuous over the nanoribbons (Figure 1d). We could also produce a single or multiple strands of free-standing BP nanoribbons via a normal mechanical exfoliation method (Figure S5).

The atomic force microscope (AFM) image (Figures 1f and S6) revealed that the surface exhibited repetitive flat-top and sharp-valley structures. The edges of the nanoribbons were amorphized with a width of 1 nm, prominently indicating thermal melting and rapid resolidification,^19^ whereas the body regions were undamaged as observed from the magnified high-resolution BF TEM image (Figure 1g). Even though the ablation was induced by the intense laser light, the resulting nanoribbons were chemically intact, free from oxidation or doping by atomic diffusion from the substrate (Figures S7 and S8).

The origin of the laser-induced periodic-structure formation in bulk metals and semiconductors with the periodicity of ∼λ_0_/*n* (*n*: an effective surface refractive index) has been generally attributed to the interference between the incident and scattered light at the surface. Periodic structures with higher spatial frequency were also found with spacing much less than λ_0_ and the interference scale. In the literature, several mechanisms have been proposed to explain the higher spatial frequency structures such as harmonic generation^24^ and surface plasmon polaritons.^25^ In our case, the high-harmonic generation is unlikely, as it is generally inefficient for higher orders, approximately tenth for our case; λ_0_ was not the exact integer multiples of the periodicity of linear structures either. In addition, the surface plasmon polariton was excluded based on its wavelength scale and wave traces. In contrast, MI caused by the intense pulsed light can develop highly ordered structures by the filamentation of the localized light in a Kerr active medium of BP. Moreover, the BP flake possessing an inversion symmetry acts as an excellent host for the nonlinear conversion of electromagnetic waves with a nonvanishing third-order susceptibility, χ^(3)^.^26^ Therefore, the Maxwell’s equation for transverse electric waves propagating along the in-plane direction can be solved to derive the MI periodicity (λ_MI_) as follows (refer to Supplementary Note 1 for details):
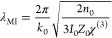
1where *k*_0_, *n*_0_, *I*_0_, and *Z*_0_ denote the wavenumber of the incident light, refractive index of BP, peak power density inside the BP flake, and vacuum impedance, respectively. In particular, *I*_0_ is on the order of 10^15^ W/m^2^, and the reported effective third-order susceptibility, χ^(3)^, is approximately 10^–16^ m^2^/V^2^;^27^ thus, λ_MI_ is computed as ∼50 nm, in agreement with our experimental observation. The 2D MI electric fields generated along the surface with the linearly polarized light were simulated using the finite difference time domain method as presented in Figures 2a–d. As the laser fluence increased from 30 to 90 mJ/cm^2^, the simulation results revealed that the partially incoherent light on the BP surface spatially disintegrated into the self-trapped arrays of the optical fields with its grating vector parallel to the polarization of the incident light like the pattern formation reported in strong nonlinear media.^28−30^ In BP, the periodic ablation followed the output patterns in the field intensity profiles. Other 2D materials with similar χ^(3)^ underwent nonuniform periodic ablation under the same irradiation condition (Figures S9 and S10). This indicates that other material properties such as thermal conductivity or sublimation/melting temperature were involved in the formation of well-defined nanostructures.

**Figure 2:**
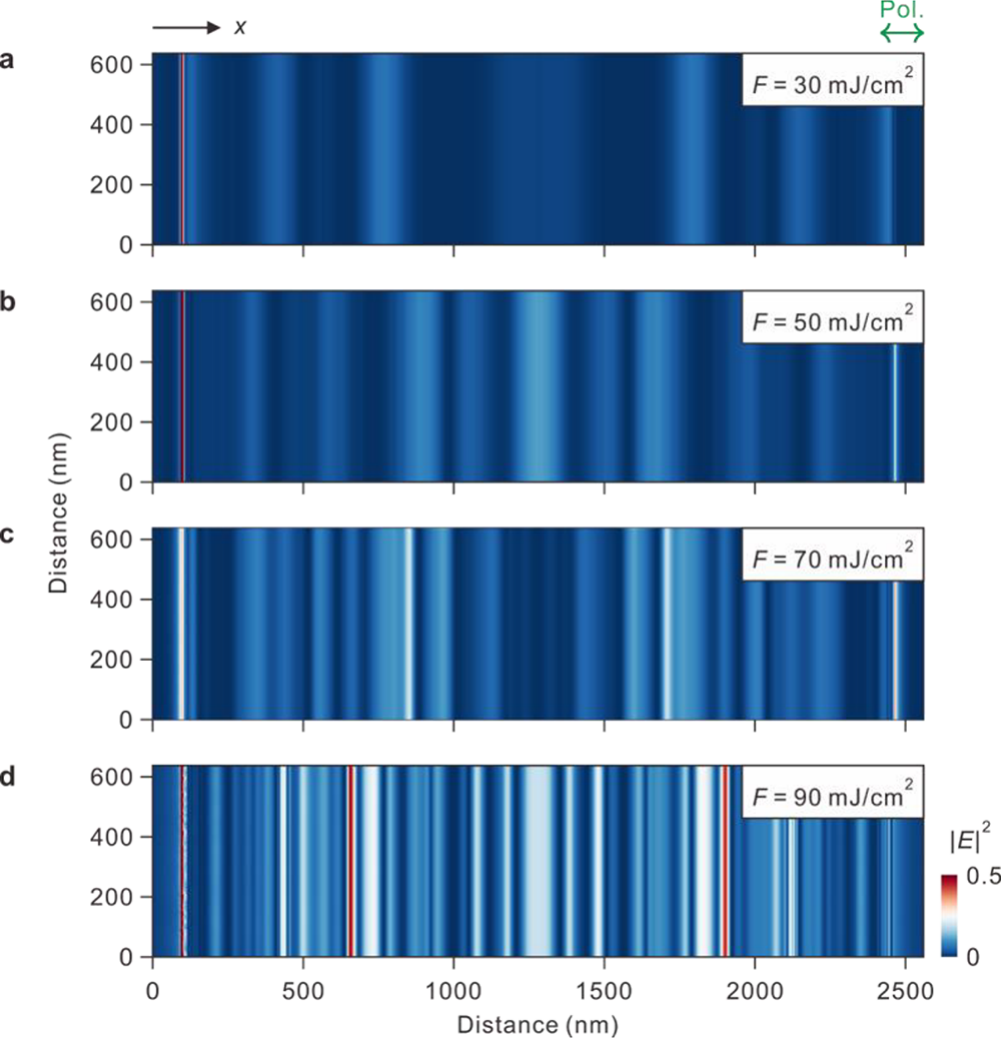
Simulation of MI electric fields in BP. (a–d) 2D field intensity (|*E*|^2^) profiles of self-trapped light owing to MI on BP surface. Laser fluences were set to 30, 50, 70, and 90 mJ/cm^2^ from panels a to d. Incident polarization was set as horizontal for all panels. Periodic boundary condition was used in vertical direction to simulate the edge-reflection free region, whereas the finite size effect was included in horizontal direction. This vividly reflects the effects of both MI and SW.

When the edge of the flake (or the truncated interface to the substrate) was impinged by the intense laser pulse, a group of nanoribbons or grooves emerged with a spacing of approximately 350 nm, as presented in Figures 3a,b. This longer periodicity was ascribed to the surface wave (SW) which launched at the flake edge and propagated away from it.^31^ As schematically illustrated in Figure 3c, the dual spatial frequency developed where the superposition of the MI fields and SW exceeded the ablation threshold. The periodic structure in Figure 3a developed when the angle between the propagation direction of the MI fields and SW is parallel (θ = 0°). In case 0° < θ < 90°, the linear patterns were uniformly disintegrated into diagonal arrays of short linear grooves (Figure 3b) as simulated in Figure 3d. Moreover, discontinuous groove arrays were formed in case the SW perpendicularly propagated to the MI fields ( = 90°) (Figure 3e). This was well reproduced in the simulated MI electric fields by employing the edge reflection (Figure 3f). This finding establishes that controlling the light polarization with respect to the orientation of the flake edge can produce various dual-frequency patterns. In case SW was annihilated by removing the edge, a well-defined arrays of nanoribbons could develop, as displayed in Figures 1d and S11.

**Figure 3:**
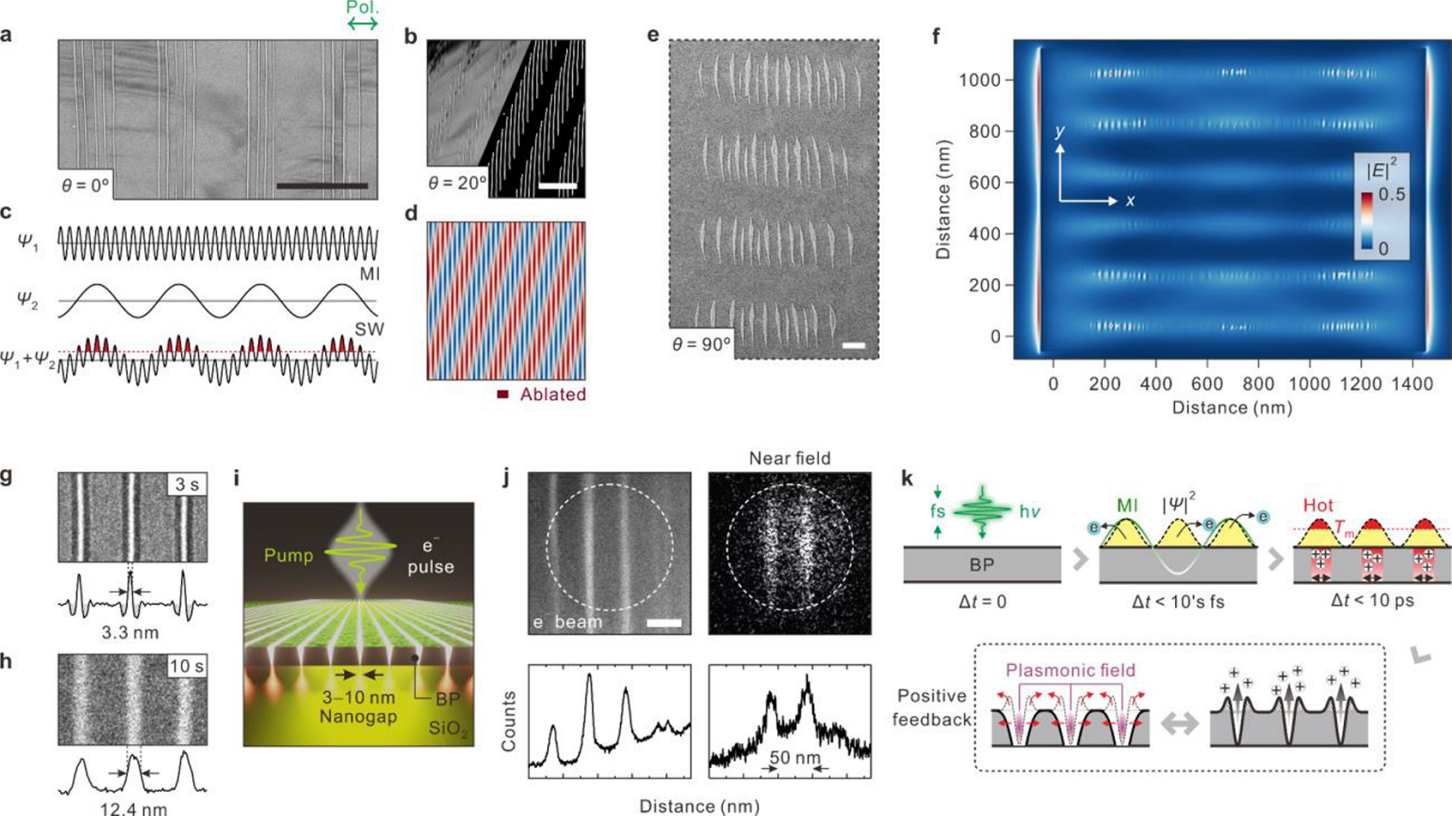
Formation mechanism of periodic BP nanogrooves. (a, b) Bright-field TEM images of dual spatial frequency nanoribbon arrays for sample 4 of panel a and sample 2 for panel b. Angles (θ) between propagation direction of SW and MI fields were 0° and 20° in panels a and b, respectively. Binary image on the right-hand side of panel b is for improved visualization of periodic structures. Scale bars: 500 nm. (c) Schematic interference between the MI field and SW. Electromagnetic fields of SW and MI constructively interfered, electron temperature increased over ablation threshold, and grooves were produced. (d) Interference simulation for the propagating SW over the MI fields in panel b. (e) Bright-field TEM image of periodic structure proximate to flake edge (sample 5). θ is 90°. Scale bar: 100 nm. (f) Simulated 2D field intensity profile of MI on finite-sized BP surface. The fluence was set to 90 mJ/cm^2^. (g, h) Bright-field TEM images and intensity profiles of nanoribbon arrays under light exposure for 3 s (sample 4) and 10 s (sample 1), respectively. (i) Illustrative view on near-field imaging of BP nanogap upon pulsed irradiation with parallel polarization to the grating vector. (j) PINEM imaging of BP nanogap (sample 6). Left, top: bright-field TEM image of BP nanogap. Right, top: energy-filtered PINEM image captured at time zero. Bottom panels: vertically averaged intensity profiles for each image. Scale bar: 50 nm. (k) Mechanism of periodic ablation by MI. When ultrashort laser pulses are introduced, MI is created at the BP surface and electrons are heated. Under continuous illumination, electrons with high thermal energy are ejected, and the remaining positive ions are condensed at the surface. When heat is accumulated and the local temperature reaches the melting point of BP, the local mass finally ablates by Coulomb explosion of the hot positive ions. The repulsive force of the positive ions repels the mass out of the way when they are released, and the mass is deposited to the sides of each gap. The positive feedback between the continuous irradiation and the ablated structure with enhanced plasmonic field at the nanogap then further widens the gap.

The width of the grooves ranged from 3 to ∼10 nm. At the initial stage of forming linear periodic grooves under the intense light (*N* = 3000), the mass along the sharp, periodic linear fields was removed and locally deposited as amorphous P (4–5 nm wide) aside each groove with 3 nm gap (Figure 3g). The area-integrated estimation of the amount of deposited mass was approximately equal to that of the mass removed along the grooves (Figure S12). Prolonged illumination sublimated the deposited mass at both sides of the grooves by positive feedback (see below) and increased the gap to 12 nm (Figure 3h). The width was approximately the span of the amorphous P walls including the vacuum grooves at the initial stage.

Initially, the intense femtosecond pulses caused rapid excitation of electrons in the material along the spatially modulated optical fields within the pulse duration. The redistribution of this energy softened atomic bonds and consequently destabilized the crystal lattice. Photoactivated ablation processes followed the liquefaction along the intense optical field, wherein the local temperature hardly exceeded the melting point of BP (600–1000 °C).^32,33^ The process was insensitive to the laser pulse duration up to 10 ps (Figure S13), which is the typical time scale of the electron–phonon and phonon–phonon scatter and implies that the thermal melting precedes the ablation. According to the 1D diffusion model, *L* = (*tD*)^1/2^, the heat diffusion length (*L*) for 10 ps (*t*) at 600 °C with the reported thermal diffusivity (*D* = 5.3 × 10^–6^ m^2^/s)^34,35^ was estimated as approximately 7 nm, which represents the typical scale of the groove width. The driving force to repel the liquefied P toward the opposite walls was potentially caused by the Coulomb explosion of positively charged mass following the electron ejection along the extreme electric fields during irradiation.^36,37^ It is rational to consider the emergence of confined plasmonic fields along the grooves, which act as nanogaps, and thus are self-consistently optimal for plasmon excitation. Synergistically, the nanogap-induced plasmonic field may facilitate mass ablation along the grooves and further widen the gap. The field confinement at the BP nanogap was verified using photon-induced near-field electron microscopy (PINEM) (Figures 3i,j).^38^ At a much lower fluence of 30 mJ/cm^2^, the confined near fields at the nanogaps were successfully imaged (Figure S14). Therefore, under consecutive pulse irradiation, the corrugated surface provided favorable conditions for positive feedback to facilitate the ablation until the nanogap effect persisted. The summary of the tailoring mechanism is illustrated in Figure 3k.

“Scissoring” a BP membrane with light was filmed via *in situ* measurements (Movie S2). The representative snapshots are illustrated in Figure 4 (extracted from Movie S2). At *F* = 96 mJ/cm^2^, the nanogrooves grew for a few minutes (*N* ≈ 10^5^). As displayed in Figure 4, when the SW and MI fields propagated in parallel to each other (θ = 0°), the two independently growing upper and lower grooves were eventually connected to a single line, reflecting that they shared a common MI field, which laterally spanned several micrometer dimensions on the surface. Note that line 1 in Figure 4, i.e., the middle groove in the field of view, developed first owing to the most intense spatial overlap between the SW and MI. Interestingly, the growth speeds of the lines were observed to vary. This local difference indicates the strong reliance of field-enhanced nanoshaping on the surface quality and topography.

**Figure 4:**
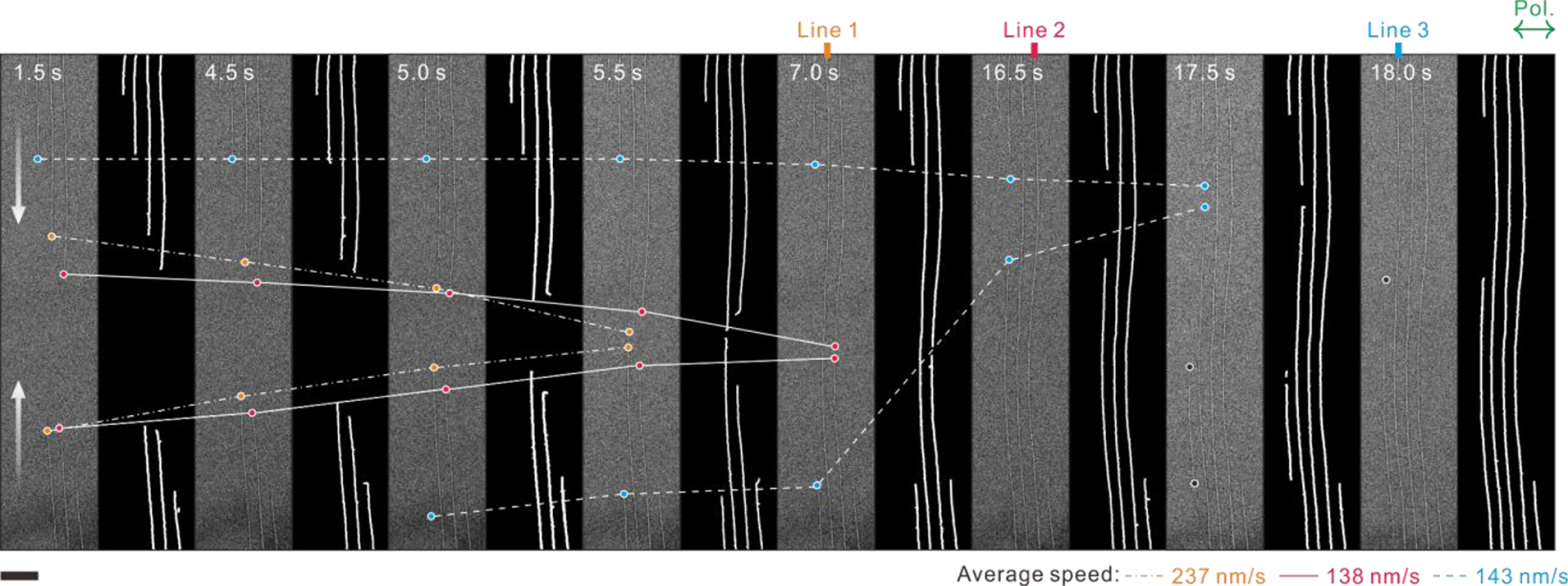
Real-time growth of BP nanoribbons. *In situ* monitoring of nanoribbon growth (sample 2). In case MI fields and SW propagated parallel to each other, nanogrooves growing from upper and lower regions were finally connected to form nanoribbons, which imply that their common origin is the MI fields, which was regularly distributed over a wide region. Binary images for TEM images are presented at the right-hand side of corresponding TEM images for improved visualization. Three grooves, lines 1–3, have varying average growth speeds as noted below the panels. Scale bar: 200 nm. Incident polarization was set to be horizontal.

The width of nanoribbons depends on the wavelength of the incident light (Figure S15) and sample thickness (Figure S16), signifying the variability of tailoring matter in size. A width of 25 nm was observed occasionally with the irradiation of 515 nm (Figure S17). We also demonstrate that BP flakes can be sculpted in any direction in a reproducible manner with a precision of few nanometers by controlling the light polarization. The insensitivity of periodic ablation to the strong optical anisotropy of BP results from the effective balance between the anisotropic absorbance and cohesive energy along the AC and ZZ axes. When BP is irradiated with AC-polarized light, the absorption is larger because of the larger extinction coefficient along the AC axis in the visible spectral region (AC: ∼1.2; ZZ: ∼0.5).^39,40^ However, when electromagnetic fields of MI are aligned along the ZZ axis, higher cohesive energy along the ZZ axis (AC: 2.93 eV; ZZ: 3.03 eV)^41^ impedes bond breaking. In the opposite case with ZZ-polarized illumination, the smaller cohesive energy along the AC axis is canceled by smaller absorption, which in turn poses similar ablation threshold as the former.

As portrayed in Figures 5a and S18, nanocubes or cuboids were formed when the two orthogonally polarized light were sequentially introduced regardless of the order; refer to Movie S3 for the real-time formation. Once nanoribbons were formed, the successive illumination—with polarization perpendicular to the preceding one—cut each nanoribbon along the short axis. The in-plane aspect ratio of the cuboids confirmed that the optical fields of MI relied on both the physical and optical properties of the materials. The formation of the initial nanostructures upon irradiation accompanied changes in surface morphology and refractive indices that altered the distribution, i.e., spatial period, of the induced optical fields therein upon the successive irradiation, aided by the positive feedback from the initial structure. For instance, as shown in Figure 5a, the first irradiation produced 50 ± 5 nm wide nanoribbons, whereas the succeeding irradiation with perpendicular polarization exhibited a larger periodicity of 60 ± 7 nm with the cut being discontinuous between the adjacent ribbons.

**Figure 5:**
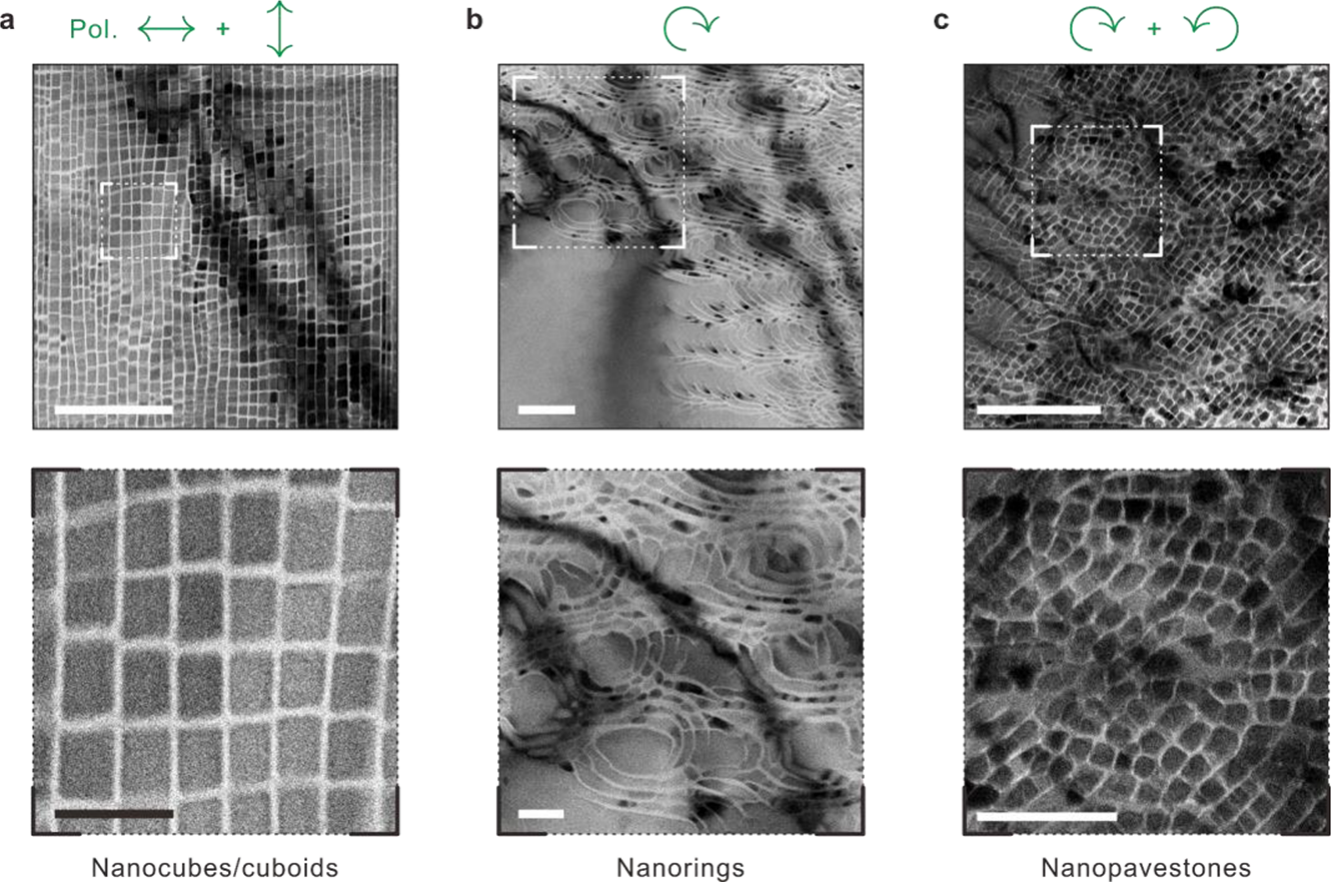
BP nanosculptures. (a–c) High- and low-magnification bright-field TEM images of nanosculptures (sample 1). (a) Sequential combination of two orthogonal light polarizations yields nanocubes/cuboids. (b) Nanorings with ablated curvatures by applying circularly polarized light (counterclockwise). (c) Randomly distributed nanopavestones. Both clockwise and counterclockwise circularly polarized light were serially introduced. Scale bars: 500 and 200 nm for the upper and lower panels, respectively.

In case the incident light was circularly polarized, concentric ring-like structures developed comprising three to four shells with in-between spacings of 50 ± 10 nm (Figure 5b); the real-time formation is presented in Movie S4. This is reasonable because the fine-spaced grooves grew normal to the light polarization (details in Figure S19). The distance among the centers of the rings was approximately 350 nm arising from the interference with SW. This observation was reproduced by the MI simulation with incident circularly polarized light (Figure S20). Furthermore, “nanopavestones” were created when both clockwise and counterclockwise polarized light were used sequentially, regardless of the order. They were organized into azimuthally curved structures, resembling Roman circular pavestones (Figure 5c). This highlights a unique freedom in designing various highly ordered BP architectures in 2D over a large area.

In this study, we delivered promises of exploiting the optical nonlinearity of BP upon photoexcitation, which focuses light into the diffraction-limit-broken regime to produce highly confined local optical fields and nanoribbons. In particular, variable nanostructures were created along the footprint of the intense optical fields over a microscale area. As a demonstration of utilizing MI to transform the 2D matter into lower dimensions, the BP nanosculptures were tailored by the appropriate selection of the incident light property. Upon capturing the evolution of the nanosculptures under intense light in real space and time, we could understand phenomena involving strong light–matter interactions and characterize the mechanism of tailoring 2D matter.

Our bottom-up approach is distinguished from EBL or other techniques of top-down processes in both technical and scientific points of view. Understanding the underlying physics of the optical spatial solitons especially at low dimensions and obtaining controllability over these strong nonlinear phenomena would offer fresh insights into light–matter interactions. The best EBL uses a focused electron beam to reach spatial precision down to a few nanometers,^42,43^ but the beam needs to raster scan a resist to inscribe shapes on it, and this point-by-point, unparalleled exposure fundamentally limits writing speeds. In this demonstration, the self-organized strong optical field induced by the laser light realizes wide-field simultaneous nanostructuring over a several micrometer area. The nanostructured area would be even extended by orders of magnitude if we can apply optical pulses of the orders-of-magnitude large diameter with the same fluence. With controlling the polarization of the laser light, it is made possible tailoring the nanoribbons along AC, ZZ, and uniquely in-between.

## Methods

### Sample Preparation

The BP TEM specimens were prepared using the PDMS-based dry transfer method. In particular, the PDMS films were fabricated using a 10:1 (w/w) prepolymer and curing agent mixture (Sylgard 184, Dow Corning Co.). Additionally, the BP flakes (purchased from Smart Elements) were exfoliated on the PDMS substrate using the mechanical exfoliation method. The thin BP flakes were identified using an optical microscope under the transmission mode, and the sample thickness was estimated using the optical transmittance.^44^ Thereafter, the identified thin BP flakes were transferred to TEM grids with a SiO_2_ substrate (21532-10, Ted Pella) and Si_3_N_4_ substrate (purchased from Norcada Inc.) using the optical microscope equipped with a micromanipulator. Subsequently, the BP surface degradation was minimized by conducting sample fabrication and optical characterization inside a nitrogen-filled glovebox at an oxygen content of <0.2 ppm.

### Instrumentation

A 200 kV TEM (JEM-2100, Jeol) was modified to host a port and obtain an optical access to the specimen, which comprised a quartz window and an aluminum mirror assembly.^45^ The specimen was excited using an ytterbium-based amplifier (s-Pulse HP, Amplitude Systèmes) with a repetition rate of 1 kHz and a pulse duration ranging from 550 fs to 10 ps. In particular, the femtosecond laser output was frequency-doubled or -quadrupled to 515 and 257 nm, respectively, and incident onto the specimen. The specimen was illuminated with a focused excitation beam of full width at half-maximum (FWHM) of 30 μm and an angle of incidence of 3–4°. The excitation polarization was varied by placing zero-order half-/quarter-waveplates before the entrance to the optical window.

Consequently, the resulting micrographs and diffractograms were captured using a CMOS (complementary metal–oxide–semiconductor)-based retractable direct electron detector (K2 Summit, Gatan), which directly detected incoming electrons without requiring a scintillator. This detection scheme significantly reduced the point spread function and improved the detective quantum efficiency at high spatial frequencies and low-dose contrasts.^23^ Moreover, a high frame rate and fast detection algorithm (“dose fractionation” mode) could minimize the counting at more than one electron per pixel, thereby eliminating the Landau noise and reducing the coincidence loss. For steady-state and time-resolved electron-energy-loss (EEL) spectra and energy-filtered images (PINEM) measurements, a CCD camera (US4000, Gatan) attached to the end of a postcolumn type imaging filter (GIF Quantum SE, Gatan) was used with the direct detector retrieved from the optical axis. High-resolution EEL spectra were obtained by an aberration-corrected TEM (JEM-ARM300F, Jeol).

## Supporting Information Available

Simulation details of modulation instability in BP (Supplementary Note 1); optical images of BP samples studied in the current work (Figure S1); sample information and laser fluence employed in the experiments (Table S1); low-magnification BF TEM images of patterned BP flakes AFM image (Figure S2); dependence of periodic ablation on the crystalline axis of BP (Figure S3); dislocation features along the periodically ablated lines (Figure S4); optical microscopy image of exfoliated nanoribbons (Figure S5); atomic force microscopy image of BP nanoribbons (Figure S6); EEL spectra of BP nanoribbons (Figures S7, S8); periodic ablation in other thin materials (Figure S9); tests with other anisotropic 2D materials (Figure S10); nanoribbon formation with and without edge effects (Figure S11); area-integrated estimation of nanogroove (Figure S12); pulse-duration dependence of nanoribbon formation (Figure S13); PINEM imaging of field confinement at BP nanogaps (Figure S14); dark-field TEM image of periodically ablated BP upon ultrashort UV (257 nm) pulse irradiation (Figure S15); thickness dependence of nanoribbons (Figure S16); spatial frequency doubling of nanoribbons (Figure S17); tailoring nanocubes/cuboids by successive illumination of two orthogonally polarized light (Figure S18); mechanism of BP nanoring formation with circularly polarized light (Figure S19); MI simulation of electric fields on BP upon irradiation with circularly polarized light (Figure S20); captions for Movies S1 to S4 (PDF)
Movie S1 (MP4)
Movie S2 (MP4)
Movie S3 (MP4)
Movie S4 (MP4)
