# Supplementary material for: Tailoring Two-Dimensional Matter Using Strong Light–Matter Interactions
Source: Nano Lett. 2023 Mar 6;23(8):3645–52. doi: 10.1021/acs.nanolett.2c04467 (PMC10141415; doi:10.1021/acs.nanolett.2c04467)
Supplement: Supplementary file 1 — nl2c04467_si_001.pdf [file nl2c04467_si_001.pdf]

Supplementary Information for

Tailoring two-dimensional matter using strong light-matter interactions

Ye-Jin Kim[1,2,†], Yangjin Lee[3,4], WonJae Choi[5], Myeongjin Jang[3,4], Won-Woo Park[1], Kwanpyo Kim[3,4], Q-Han Park[5,*], and Oh-Hoon Kwon[1,2,*]

[1] Department of Chemistry, College of Natural Sciences, Ulsan National Institute of Science and Technology (UNIST), 50 UNIST-gil, Ulsan 44919, Korea

[2] Center for Soft and Living Matter, Institute for Basic Science (IBS), 50 UNIST-gil, Ulsan 44919, Republic of Korea

[3] Department of Physics, Yonsei University, 50 Yonsei-ro, Seoul 03722, Republic of Korea

[4] Center for Nanomedicine, IBS, 50 Yonsei-ro, Seoul 03722, Republic of Korea

[5] Department of Physics, Korea University, 145 Anam-ro, Seoul 02841, Republic of Korea

* Correspondence and requests for materials should be addressed to Q-H.P. (email: qpark@korea.ac.kr) and O.-H.K. (email: ohkwon@unist.ac.kr)

† Present Address: Division of Chemistry and Chemical Engineering, California Institute of Technology, Pasadena, California 91125, U.S.A

This PDF file includes:

Note 1. Simulation of modulation instability in BP
Figs. S1 to S20
Captions to Movies 1 to 4
References

Other Supplementary videos for this manuscript include the following:

Movies 1 to 4

I. Supplementary Note 1: Simulation of modulation instability in BP

The nonlinear optical response of BP toward the intense excitation beam can be perturbatively described by including the third-order Kerr nonlinear polarization term. Therefore, the wave equation derived from Maxwell's equation based on Kerr nonlinearity can be expressed as

$$\nabla \times \nabla \times \mathbf{E} = k_0^{\ 2}\left[\varepsilon \mathbf{E} + \frac{1}{2}\chi^{(3)}\left(\left|\mathbf{E}\right|^2 \mathbf{E} + \frac{1}{2}\left(\mathbf{E}\cdot\mathbf{E}\right)\mathbf{E}^*\right)\right] . \tag{0.1}$$

Here, $\chi^{(3)}$ denotes the third-order susceptibility (for simplicity, we considered the isotropic case only) and $\mathbf{E}$, $\mathbf{H}$, k_0, and ε denote the electric field, magnetic field, vacuum wavenumber, and permittivity, respectively. As BP and SiO_2 acted as dielectric materials for the light with wavelength 515 nm, the thin BP layers supported the transverse electric (TE) guided mode in the linear regime. In presence of the third-order nonlinear terms, the TE and transverse magnetic (TM) modes were no longer separable but became mixed. However, if we assumed that the nonlinear term caused only a slow modulation over the tangential *xy*-plane of BP, a TE-like mode can be derived as

$$\mathbf{E} = \left(0, E_y\left(x, y\right), 0\right) e^{ik_z z} e^{iqx} , \tag{0.2}$$

which satisfied the slowly varying condition; $\chi^{(3)}\left|\mathbf{E}\right|^2 \ll \varepsilon$, $\left|\partial_x E_y\right| \ll \left|qE_y\right|$. This reduced the wave equation in (0.1) to the nonlinear Schrödinger-type equation[1]

$$\partial_x E_y - \frac{i}{2q}\partial_y^{\ 2} E_y = i\gamma\left|E_y\right|^2 E_y, \quad \left(\gamma = \frac{3k_0^{\ 2}\chi^{(3)}}{8q}\right), \tag{0.3}$$

where $k_0 = \omega / c$ denotes the wavenumber, and we considered the TE waves propagating along the *x*-direction.

An exact nondiffracting continuous-wave (CW) eigenmode solution to (0.3) can be readily determined to yield

$$E_y^{\text{cw}} = \sqrt{P} e^{i\gamma P x}, \quad \left(P = \frac{2I_0 Z_0}{n_0}\right), \tag{0.4}$$

where $n_0 = \sqrt{\varepsilon}$ represents the refractive index of the medium, $Z_0 = \sqrt{\mu_0 / \varepsilon_0} \approx 377\ \Omega$ expresses the vacuum impedance, and I_0 denotes the intensity of electromagnetic waves inside the medium. This nondiffracting CW mode can become unstable against small fluctuations, thereby resulting in MI. Thus, to explicitly characterize MI, we considered the perturbation ansatz as

$$E_y = \left(\sqrt{P} + a(x,y)\right)e^{i\gamma Px}, \quad \left(P \gg \left|a(x,y)\right|^2\right), \tag{0.5}$$

which satisfied the perturbative equation up to the second order in *a*,

$$\partial_x a - \frac{i}{2q}\partial_y{}^2 a = i\gamma\left(P\left(a + a^*\right) + \sqrt{P}\left(a^2 + 2aa^*\right)\right). \tag{0.6}$$

As discussed later, the second-order terms were responsible for the spatial frequency doubling MI. As a and a^* were coupled, we sought the solutions of the form,

$$a = c_1 e^{i\xi x + i\eta y} + c_2 e^{-i\xi^* x - i\eta y} + c_3 e^{2i\xi x + 2i\eta y} + c_4 e^{-2i\xi^* x - 2i\eta y} \tag{0.7}$$

with undetermined parameters complex and real η. Therefore, Equation (0.6) requires that

$$\begin{aligned}
\left(\frac{\eta^2}{2q} - \gamma P + \xi\right)c_1 - \gamma P c_2^* &= 0 \\
\left(\frac{\eta^2}{2q} - \gamma P - \xi^*\right)c_2 - \gamma P c_1^* &= 0 \\
\left(\frac{2\eta^2}{q} - \gamma P + 2\xi\right)c_3 - \gamma P c_4^* &= \gamma\sqrt{P}\left(c_1 + c_2^*\right)c_1 \\
-\gamma P c_3 + \left(\frac{2\eta^2}{q} - \gamma P - 2\xi\right)c_4^* &= \gamma\sqrt{P}\left(c_1 + c_2^*\right)c_2^*
\end{aligned}. \tag{0.8}$$

Moreover, nontrivial solutions exist for c_1 and c_2 if

$$\xi^2 = \left(\frac{\eta^2}{2q} - \gamma P\right)^2 - \gamma^2 P^2. \tag{0.9}$$

Recall that the TE-like mode in (0.2) progresses along the *x*-axis in a BP slab. We report that η describes the width of the transverse pattern in parallel to the *y*-axis, whereas complex ξ signals instability. Furthermore, the modulation instability occurred when $\xi^2 < 0$ or

$$\left(\frac{\eta^2}{2q}-\gamma P\right)^2-\gamma^2P^2<0 \quad \text{or} \quad \eta<\eta_C=\sqrt{4q\gamma P}\,. \tag{0.10}$$

Note that the maximum modulation occurred for

$$\eta=\eta_{\mathrm{M}}=\sqrt{2q\gamma P}=k_0\sqrt{\frac{3I_0Z_0\chi^{(3)}}{2n_0}}\,, \tag{0.11}$$

for which the pattern width λ_{MI} can be approximately calculated as

$$\lambda_{\mathrm{MI}}=\frac{2\pi}{\eta_{\mathrm{M}}}=\frac{2\pi}{k_0}\sqrt{\frac{2n_0}{3I_0Z_0\chi^{(3)}}}\,. \tag{0.12}$$

For a quantitative estimation, we selected the parameters

$$\begin{gathered} n_{\mathrm{zz}}=3.56+0.126i,\ n_{\mathrm{ac}}=3.29+0.428i,\ n_0\approx 3.4 \\ \frac{2\pi}{k_0}=\lambda_0=515\,\mathrm{nm},\ \ Z_0\approx 377\ \Omega,\ I_{\mathrm{laser}}^{\mathrm{Peak}}\approx 1.7\times10^{15}\ \mathrm{W/m}^2 \end{gathered}\,. \tag{0.13}$$

Unfortunately, we could not derive a well-determined $\chi^{(3)}$ value for BP under 550-fs light pulses at 515 nm. In addition, the intensity I_0 in (0.12) was not equal to the peak value $I_{\mathrm{laser}}^{\mathrm{peak}}$ of the excitation laser pulse but could be locally enhanced. If we assume $I_0\approx I_{\mathrm{laser}}^{\mathrm{peak}}$ without counting local enhancement and use a reasonable estimation of $\chi^{(3)}$, *i.e.*, $\chi^{(3)}\sim 3\times10^{-16}m^2/V^2$ 2, then $\chi^{(3)}I_0\sim 0.5\ \Omega^{-1}$, for which we could determine $\lambda_{\mathrm{MI}}\sim 50\,nm$. This was in appropriate agreement with the observed pattern.

In case the maximum modulation condition (0.11) was satisfied, we could determine all the remaining expansion coefficients

$$\eta=\sqrt{2q\gamma P},\ \ \xi=-i\gamma P,\ \ c_2^{\,*}=-ic_1,\ c_3=\frac{2-i}{6}\frac{c_1^{\,2}}{\sqrt{P}},\ \ c_4^{\,*}=\frac{-2-i}{6}\frac{c_1^{\,2}}{\sqrt{P}}\,. \tag{0.14}$$

Furthermore, we observed that the coefficients c_3 and c_4, including the measuring patterns with double spatial frequency, were suppressed by a factor $c_1\big/\sqrt{P}$ compared to c_1. However, as the modulation grew along the *x*-direction, the double spatial frequency pattern with small c_3 and

c_4 started to grow with the rate of $\exp\left(2\,\mathrm{Re}(\xi)x\right)$, which was faster than the first-order MI, and subsequently, became visible. This behavior agreed well with the experimental observations. This confirmed that the MI was the underlying physical origin of the measured patterns.

FDTD numerical calculation of MI

To derive the MI condition for BP, we assumed an instantaneous third-order nonlinear response as stated in (0.1). In the simulation, we neglected the third-harmonic terms and retained only the terms oscillating at the frequency ω, which included the contribution of molecular vibrations (Raman effect) with the fractional contribution β [3].

$$P^{(3)}(t) = \varepsilon_0 \beta \chi^{(3)} \left|E\right|^2 E + \varepsilon_0 (1-\beta)\chi^{(3)} QE \tag{0.15}$$

The delayed Raman response Q satisfied an oscillator equation

$$\frac{d^2Q}{dt^2} + 2\gamma_R \frac{dQ}{dt} + \omega_R^2 Q = \omega_R^2 \left|E\right|^2 \tag{0.16}$$

with damping parameter γ_R and Raman resonance angular frequency ω_R. Therefore, the Maxwell equation corresponding to the nonlinear medium could be expressed as

$$\nabla \times E = -\mu_0 \frac{\partial H}{\partial t}, \quad \nabla \times H = \varepsilon_\infty \frac{\partial E}{\partial t} + J\,, \tag{0.17}$$

where μ_0 denotes vacuum permeability and ε_∞ represents the background permittivity. The current J satisfied the nonlinear Lorentz model equation,

$$\begin{aligned} &\partial_t^2 J + 2\gamma \partial_t J + \omega_p^2 J = \varepsilon_0 \omega_p^2 \partial_t S, \\ &S = \chi^{(1)} E + \beta \chi^{(3)} \left|E\right|^2 E + (1-\beta)\chi^{(3)} QE \end{aligned} \tag{0.18}$$

We numerically solved the nonlinear Maxwell equation by extending the finite difference time domain method to include the nonlinear term with difference equations:

$$\begin{aligned}
&\frac{1}{dt^2}\left(J^{n+1}-2J^{n}+J^{n-1}\right)+\frac{\gamma}{2dt}\left(J^{n+1}-J^{n-1}\right)+\omega_p^2 J^{n}=\frac{\varepsilon_0\omega_p^2}{2dt}\left(S^{n+1}-S^{n-1}\right)\\
&S_k^{n}=\chi^{(1)}E_k^{n}+\left(1-\beta\right)\chi^{(3)}\left|E^{n}\right|^2 E_k^{n}+\beta\chi^{(3)}\left|Q^{n}\right|^2 E_k^{n},\\
&E_k^{n+1}=E_k^{n}-\frac{dt}{2\varepsilon_\infty}\left(J_k^{n+1}+J_k^{n}\right)+\frac{dt}{\varepsilon_\infty}(\nabla\times H)^{n+1/2}\\
&\frac{1}{dt^2}\left(Q^{n+1}-2Q^{n}+Q^{n-1}\right)+\frac{\gamma_R}{2dt}\left(Q^{n+1}-Q^{n-1}\right)+\omega_R^2 Q^{n}=\omega_R^2\left|E^{n}\right|^2
\end{aligned},\qquad (0.19)$$

which were iteratively solved using the updated equations:

$$\begin{aligned}
&Q^{n+1}=-\frac{1-\gamma_R dt/2}{1+\gamma_R dt/2}Q^{n-1}+\frac{2-\omega_R^2 dt^2}{1+\gamma_R dt/2}Q^{n}+\frac{\omega_R^2 dt^2}{1+\gamma_R dt/2}\left|E^{n}\right|^2\\
&S_k^{n}=\chi^{(1)}E_k^{n}+\left(1-\beta\right)\chi^{(3)}\left|E^{n}\right|^2 E_k^{n}+\beta\chi^{(3)}\left|Q^{n}\right|^2 E_k^{n},\\
&J_k^{n+1}=\frac{2-\omega_p^2 dt^2}{1+\gamma dt/2}J_k^{n}-\frac{1-\gamma dt/2}{1+\gamma dt/2}J_k^{n-1}+\frac{\varepsilon_0\omega_p^2 dt}{2(1+\gamma dt/2)}\left(S_k^{n+1}-S_k^{n-1}\right)\\
&E_k^{n+1}=E_k^{n}-\frac{dt}{2\varepsilon_\infty}\left(J_k^{n+1}+J_k^{n}\right)+\frac{dt}{\varepsilon_\infty}(\nabla\times H)^{n+1/2}
\end{aligned}.\qquad (0.20)$$

In the simulations, we selected the Lorentz model parameters γ,ω_p to fit the refractive index of BP, $n_{BP}=(n_{\mathrm{zz}}+n_{\mathrm{ac}})/2\approx 3.4+0.3i$, and $\chi^{(3)}\approx 5\times10^{-21}m^2/V^2$. As the measured values were unavailable for the Raman parameters, we adopted the typical values of Raman parameters for a silicon fiber[4].

II. Supplementary Figs. S1 to 20

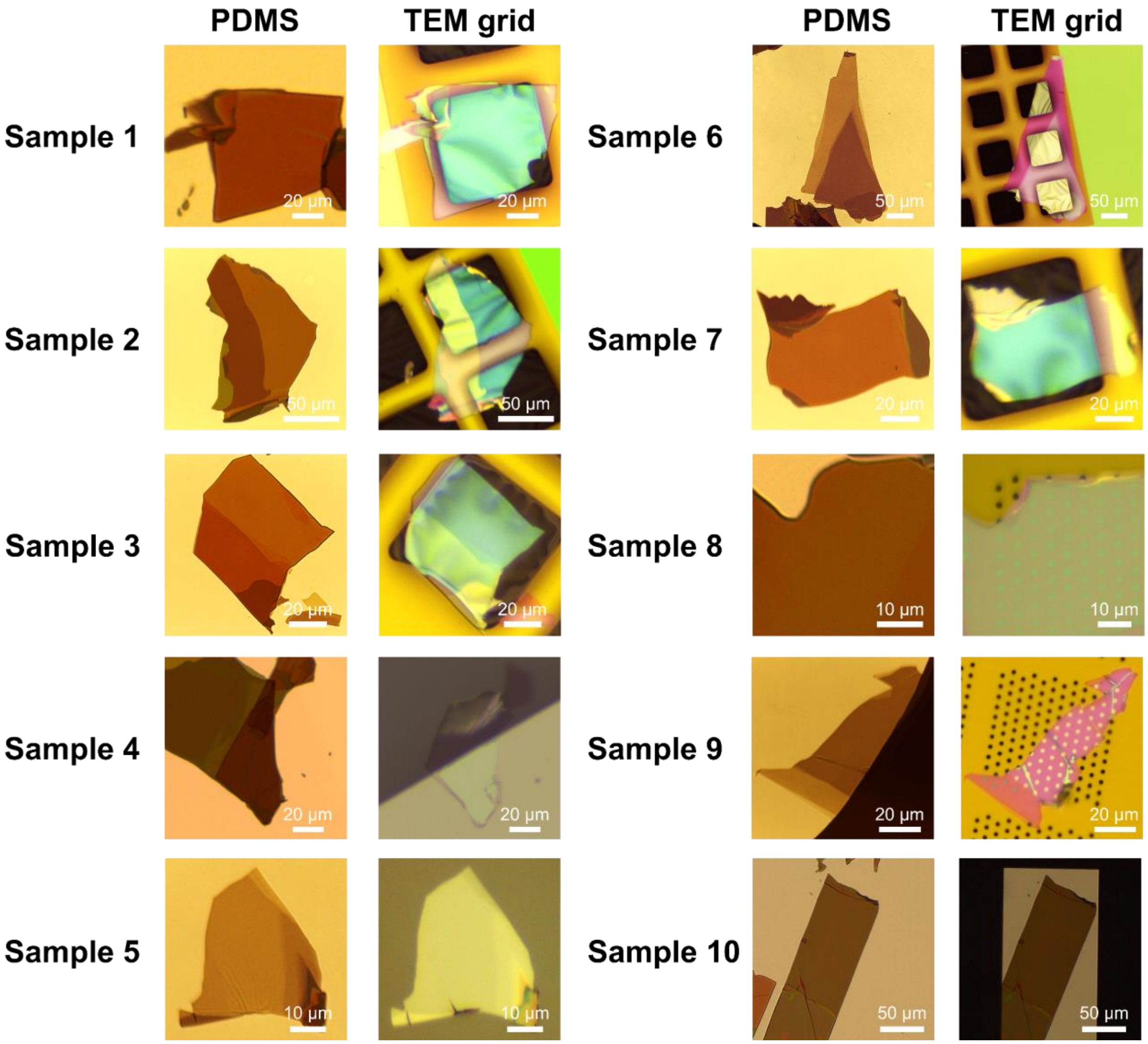

Figure S1. Optical images of BP samples studied in the current work. An optical microscope in the transmission mode was used to estimate sample thickness before transferring to TEM grids. (Left) BP flakes on PDMS films prior to transferring to the TEM grids, (right) Corresponding BP flakes transferred onto TEM grids.

Table S1. Sample information and laser fluence employed in the experiments.

Sample	Substrate	Number of BP layers	Transmittance (%)	Fluence (mJ/cm^2)	Note
1	8-nm SiO_2	30	37	90	Discussed in the main text
2	8-nm SiO_2	30	37	96	
3	8-nm SiO_2	27	42	98	
4	10-nm Si_3N_4	>30	30	94	
5	50-nm Si_3N_4	12	63	93	
6	8-nm SiO_2	15	57	95	
7	8-nm SiO_2	30	37	96	Discussed in the Supplementary Materials
8	200-nm holey Si_3N_4	28	40	95	
9	200-nm holey Si_3N_4	20	51	115	
10	8-nm SiO_2	26	45	95	

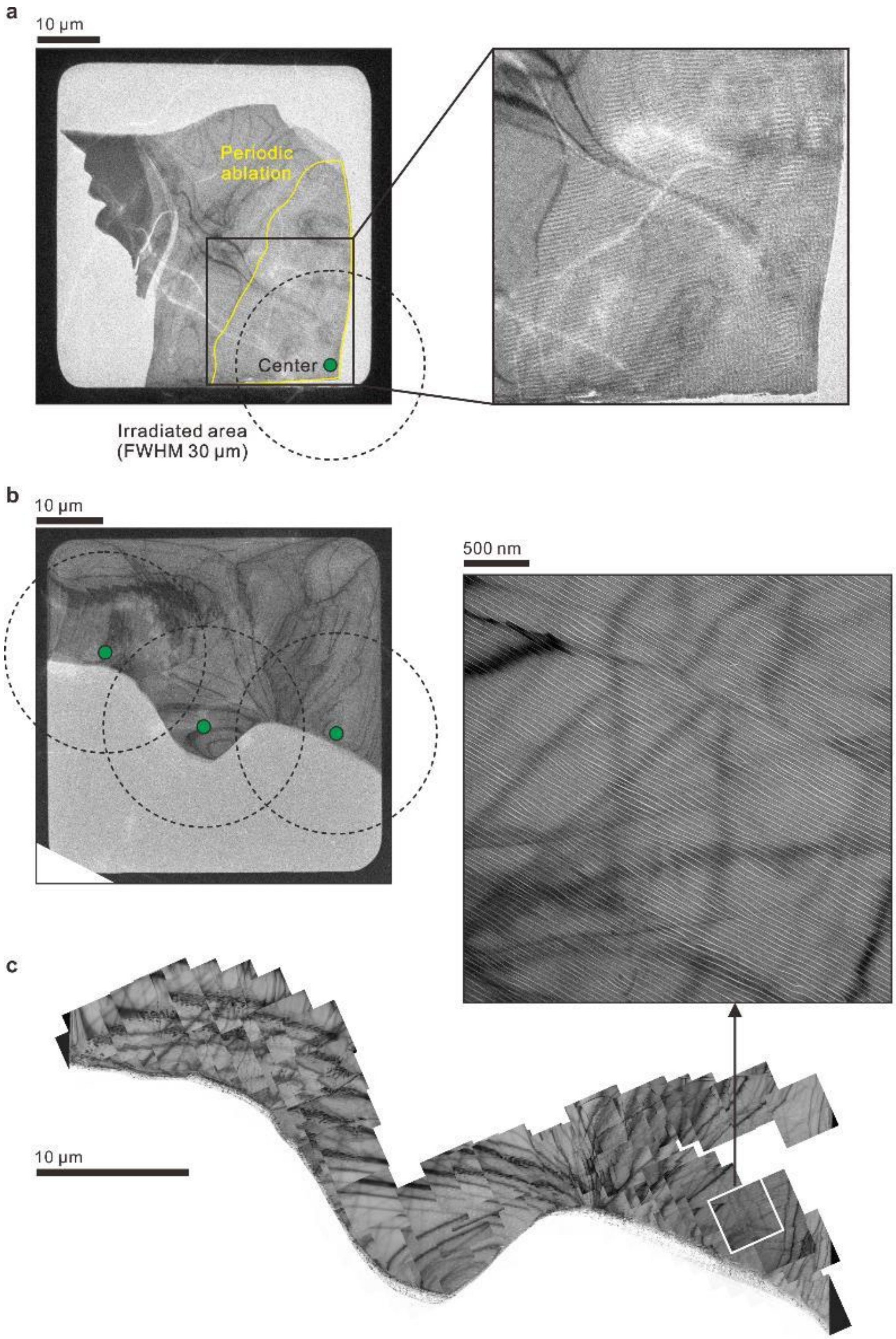

Figure S2. Low-magnification bright-field (BF) TEM images of patterned BP flakes. Centers of irradiated areas with diameters (FWHM) of 30 μm are displayed together. **a,** Sample 7. **b** and **c**, Sample 6. Panel **c** is a stitched high-magnification image of a boxed region in panel **b**. Periodic ablation occurred over a micrometer-scale area (> 10 × 10 μm^2).

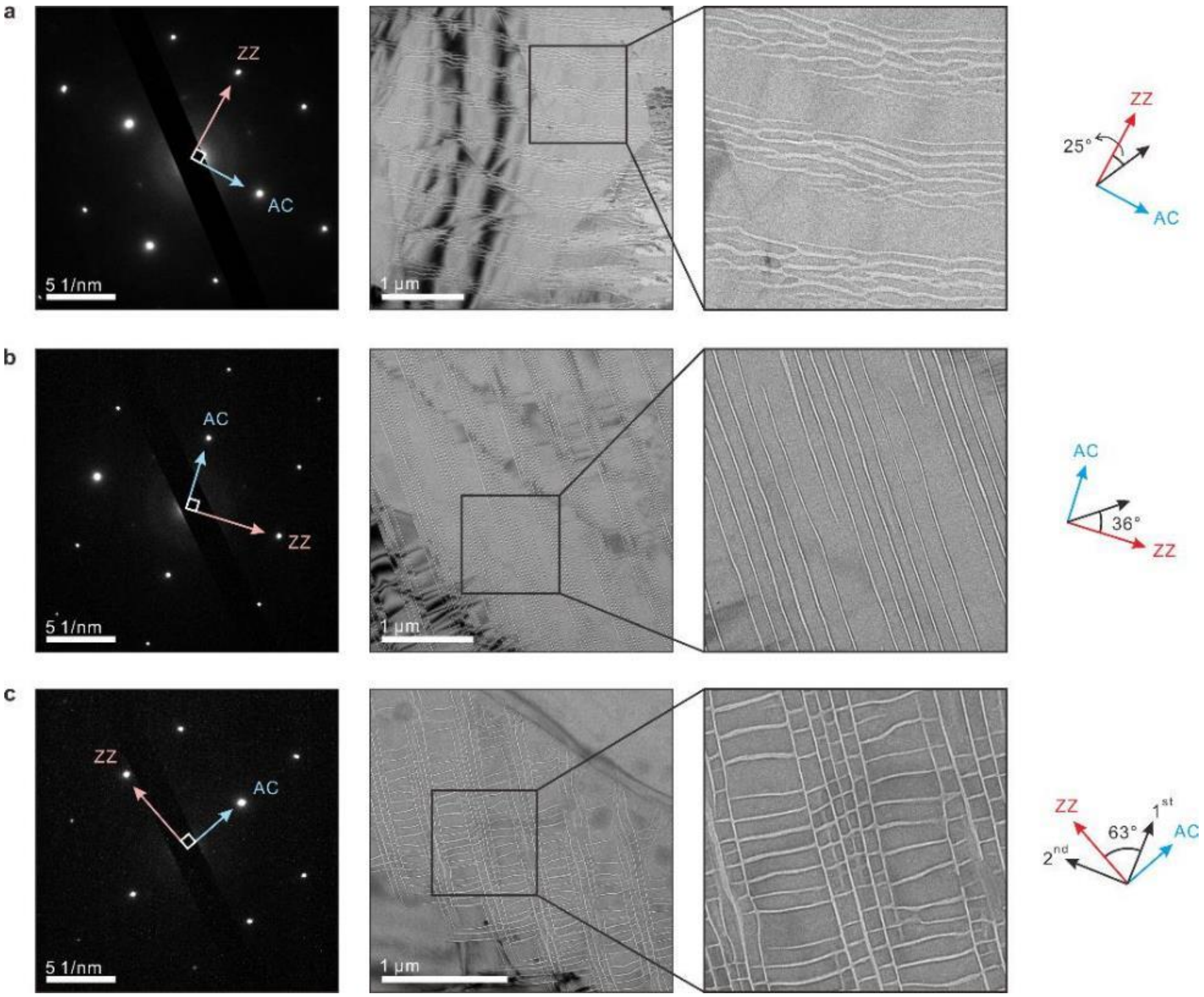

Figure S3. Dependence of periodic ablation on the crystalline axis of BP. Nanoribbons can be tailored along any directions between the AC and ZZ axes. Polarization of the incident light, *i.e.*, grating vectors of the nanoribbon arrays, are marked with black arrows between the AC and ZZ axes. **a–c,** the grating vectors directed to 25°, 36°, and 63° from the ZZ axis, respectively. For the successive irradiation in panel **c**, the polarization of the second irradiation (2nd) was perpendicular to the first one (1st). **a,** Sample 7, **b,** Sample 4, **c,** Sample 2.

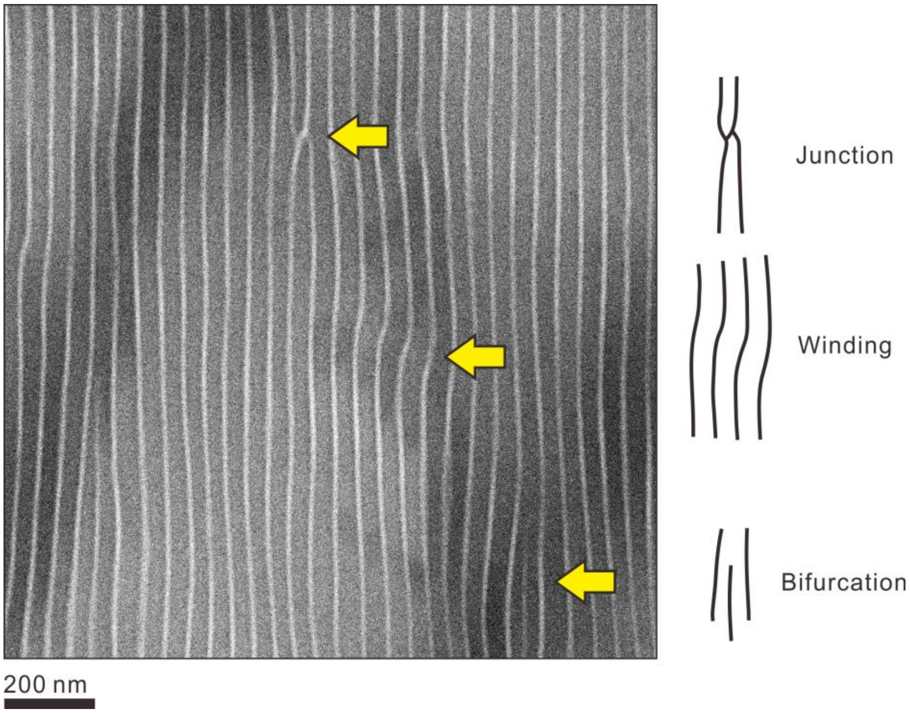

Figure S4. Dislocation features along the periodically ablated lines. Surface morphology is considered to affect local nonuniformity of the periodic ablation. In the field of view, junction, bending, and bifurcation spots were intermittently distributed. Sample 1.

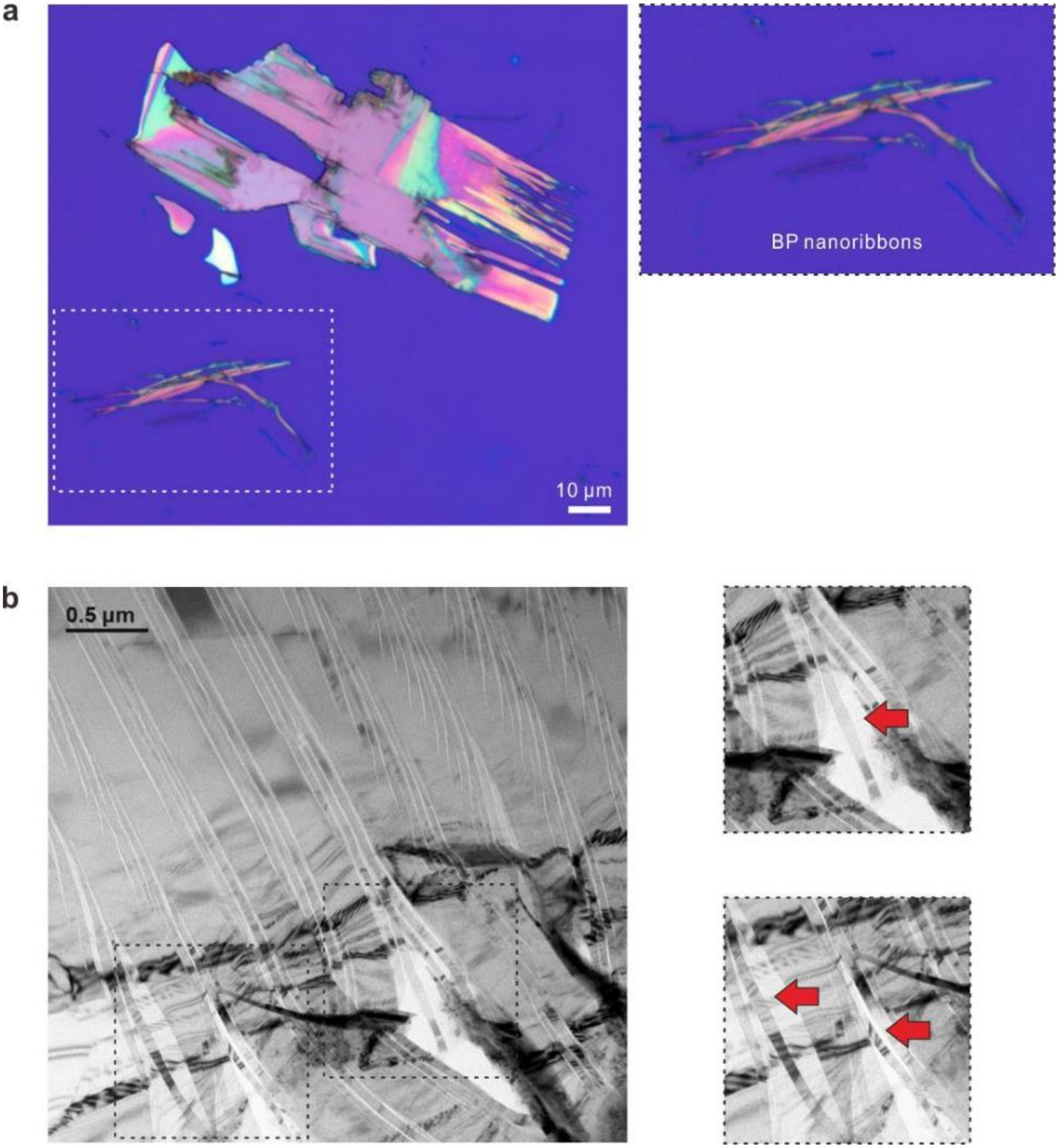

Figure S5. Optical microscope image of exfoliated nanoribbons. a, A set of single or multiple strands of BP nanoribbons (the arrays) can be separated from the original flake by mechanical exfoliation. **b,** TEM images of separated single strand nanoribbons. Both the images show the possibility of isolating single or multiple strand BP nanoribbons.

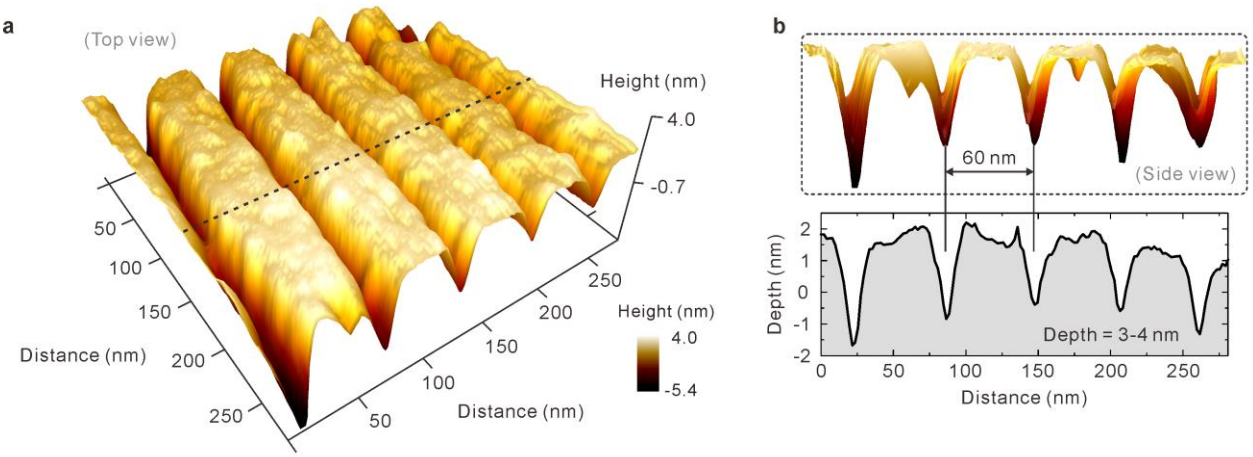

Figure S6. Atomic force microscope image of BP nanoribbons. Sample 2. **a,** 3D perspective of region of interest. **b,** Side view and height profile along dotted line in panel **a**. Average pit depth ranges from several nm to entire thickness of flakes.

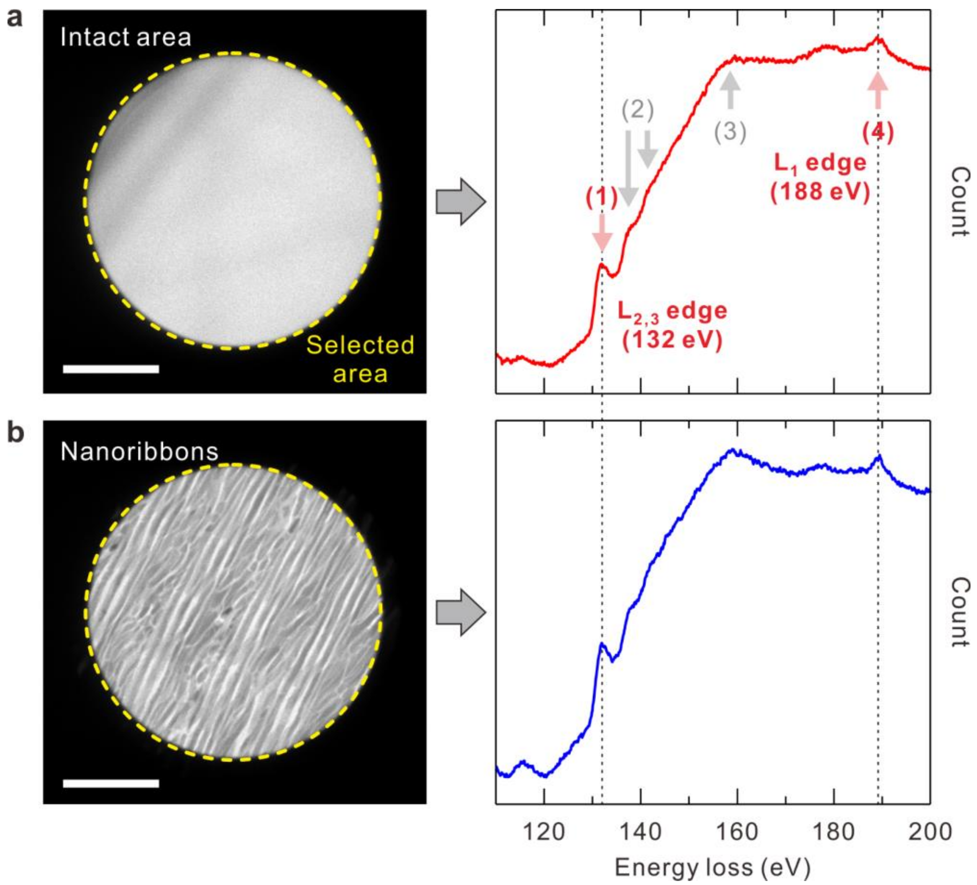

Figure S7. EEL spectra of BP nanoribbons. Bright-field TEM images (left) and EEL spectra (right) of **a,** an intact area and **b,** nanoribbons. Spectral feature in the upper panel is dominated by the core level transitions of phosphorus at 132 eV [$L_{2,3}$ edges (1)], 137 and 140 eV [2$p \rightarrow 3s$ and 3d, respectively, (2)], 158 eV [2$p \rightarrow d$ (3)], and 188 eV [L_1 edge (4)]. Sample 10. Scale bar: 500 nm.

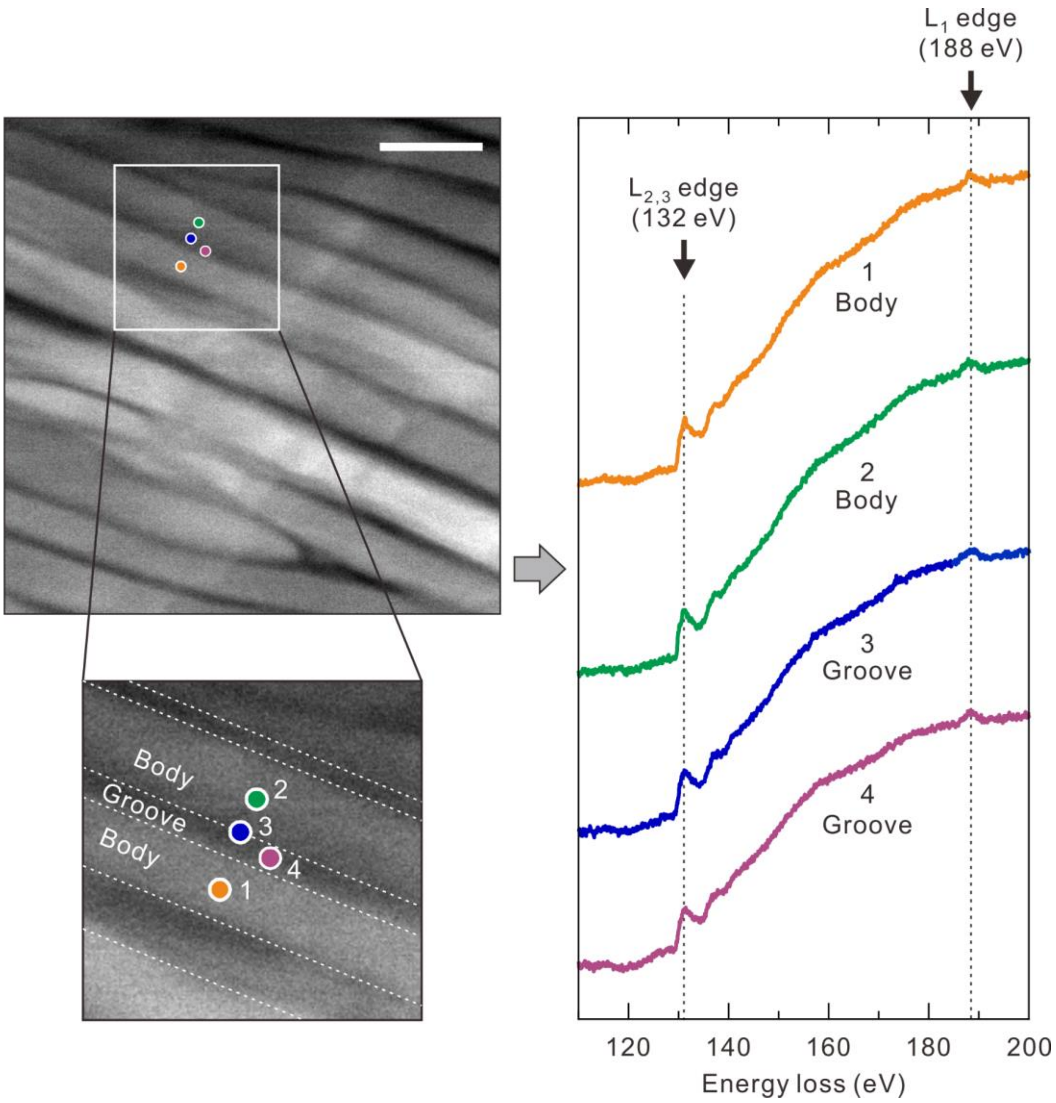

Figure S8. High-resolution EELS measurements. With an atomic-scale electron probe, spectral features at both the bodies and grooves of nanoribbons are observed to be the same. Sample 10. Scale bar: 100 nm.

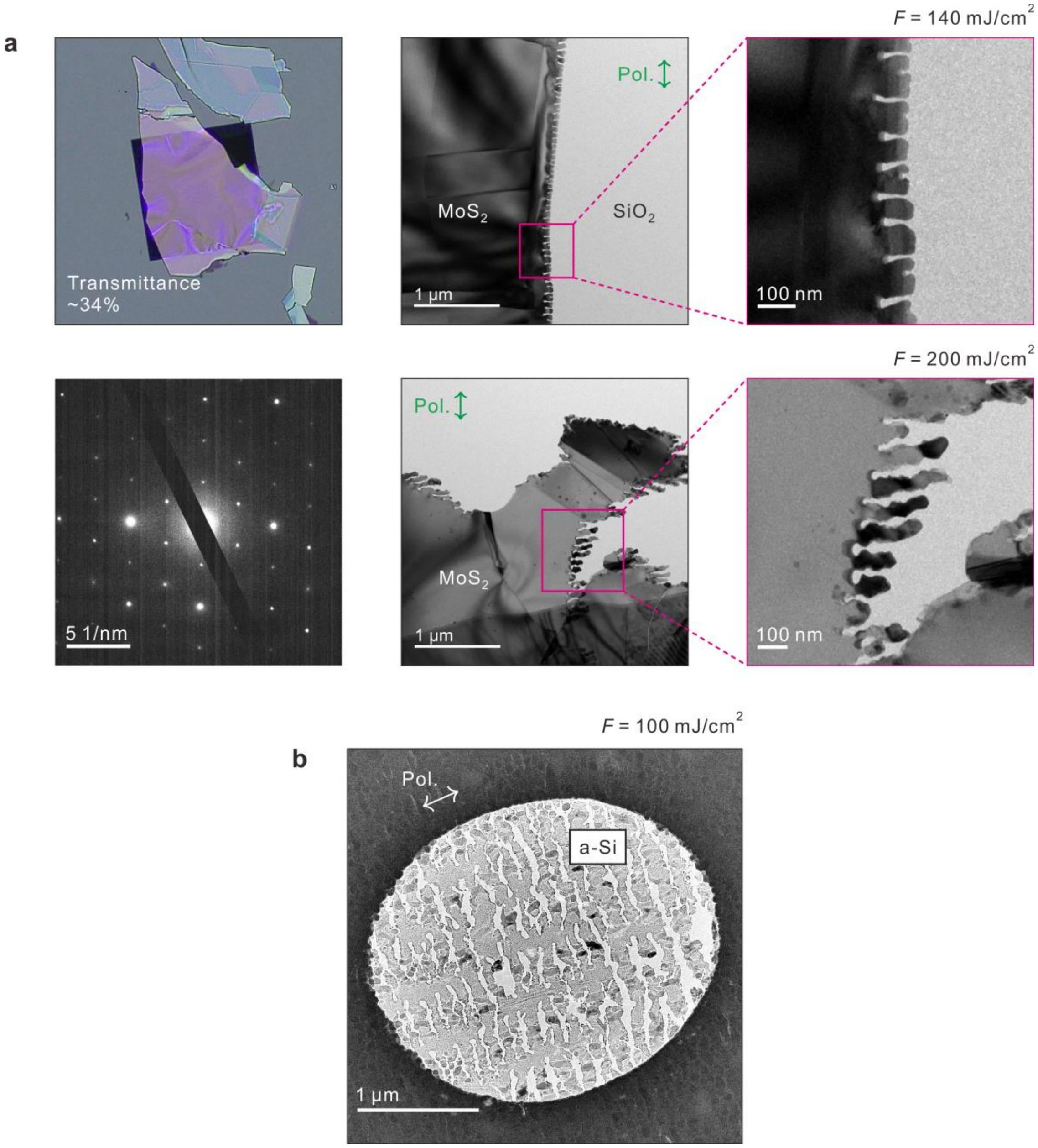

Figure S9. Periodic ablation in other thin materials. a, A molybdenum disulfide (MoS_2) flake of $\chi^{(3)}$ similar to BP (Upper left) Optical image with a transmittance of approximately 34%. (Lower left) Diffractogram. (Upper middle and right) TEM images. Upon irradiation with vertically polarized light at F = 140 mJ/cm^2 (higher fluence than that applied to BP), the flake edge was periodically ablated with periodicity of 60–70 nm similar to the case of BP. In the enlarged view, the ablated patterns and shapes were different from those formed in BP. This seems resulting from the difference in the materials' inherent properties. (Lower middle and right) TEM images. In the inner region, the flake was periodically ablated at $F \geq$ 200 mJ/cm^2. Nanoribbons did not develop in the MoS_2 flake at any fluence. **b,** Amorphous silicon (a-Si) film. At F = 100 mJ/cm^2, the film was periodically ablated, but nonuniformly and discontinuously.

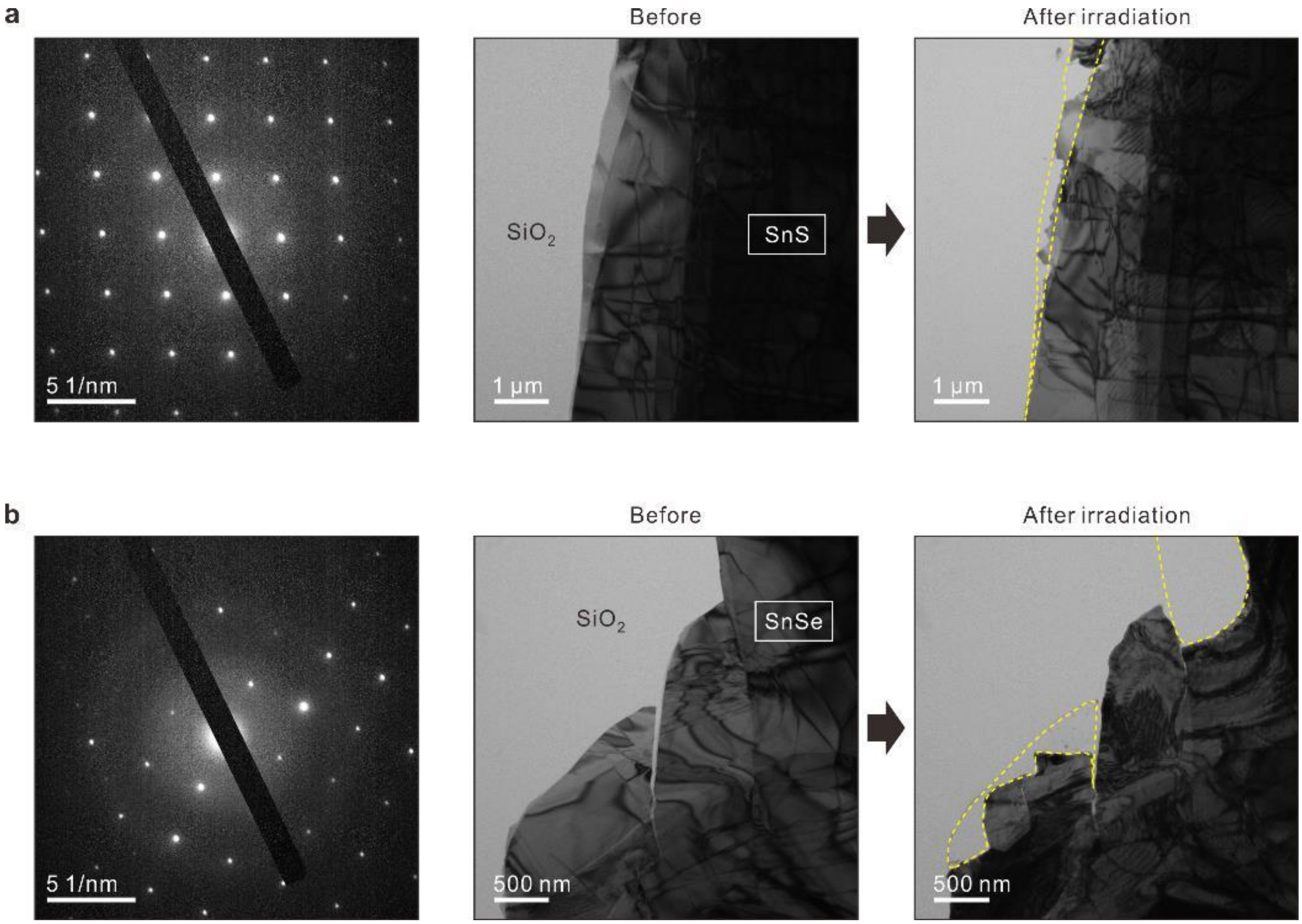

Figure S10. Tests with other anisotropic 2D materials. a, Tin sulfide (SnS) flake. Upon irradiation with linearly polarized light at F = 90 mJ/cm^2, there was no periodic ablation even at the flake edge of lower threshold, instead the flake was cracked and split into pieces (yellow dotted region). **b,** Tin selenide (SnSe) flake. At the same irradiation condition as used for BP, SnSe was also cracked into pieces (yellow dotted region). The observation signifies the fact that the formation of the well-defined array of nanoribbons in BP is not attributed to the structural anisotropy, but nonlinear optical property of materials inducing MI is the main factor giving rise to the periodic ablation.

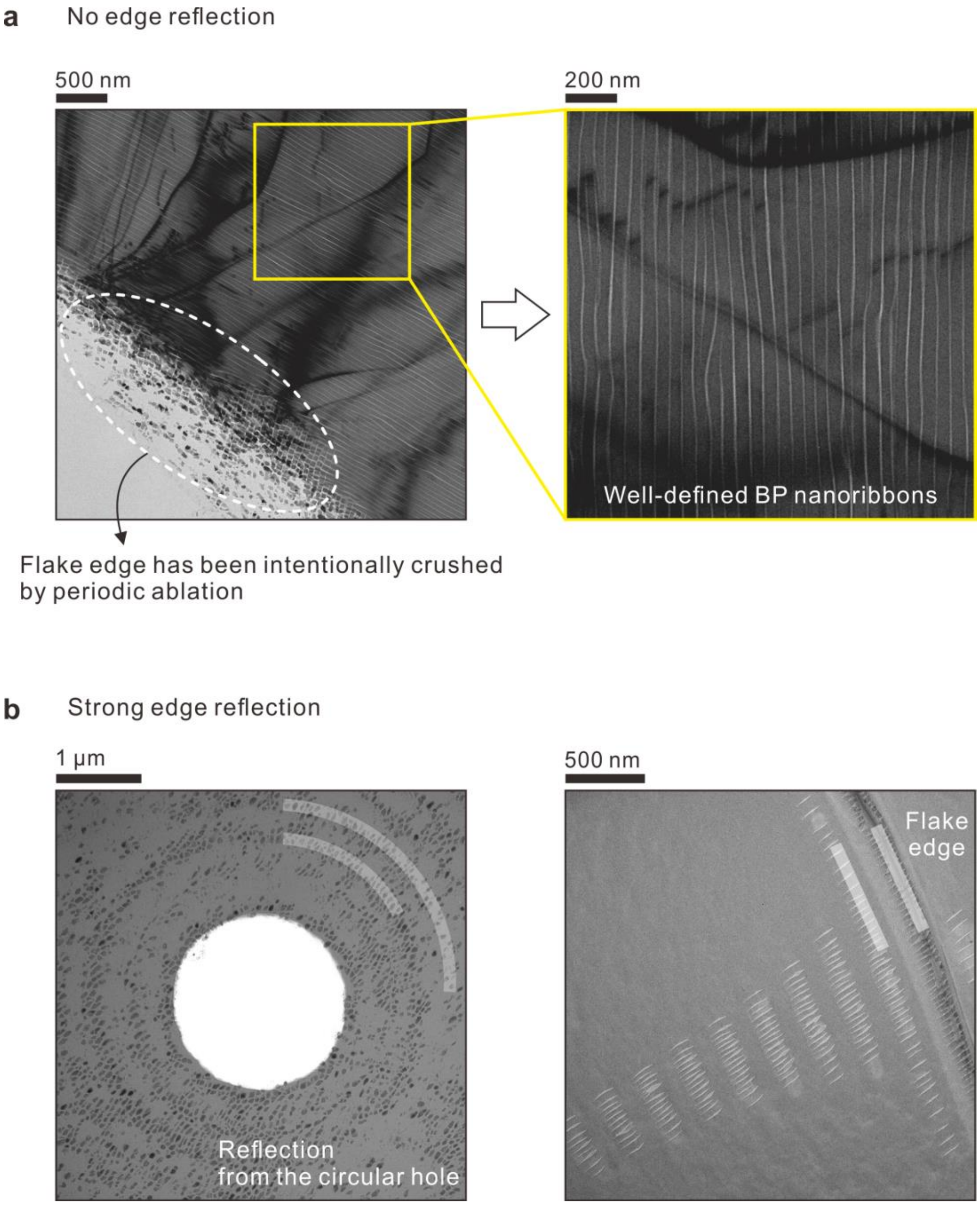

Figure S11. Nanoribbon formation with and without edge effects. a, Without edge effects. In case a flake edge was crushed or damaged, SW reflection from the edge was annihilated (absent?) resulting in the development of uniform nanoribbon arrays. Sample 6. **b,** In case structural obstacles or flake edges were intact within the irradiation area, dual-frequency periodic structures with both the 50 and 350 nm spacings were simultaneously formed. (Left) Sample 8, (right) Sample 4.

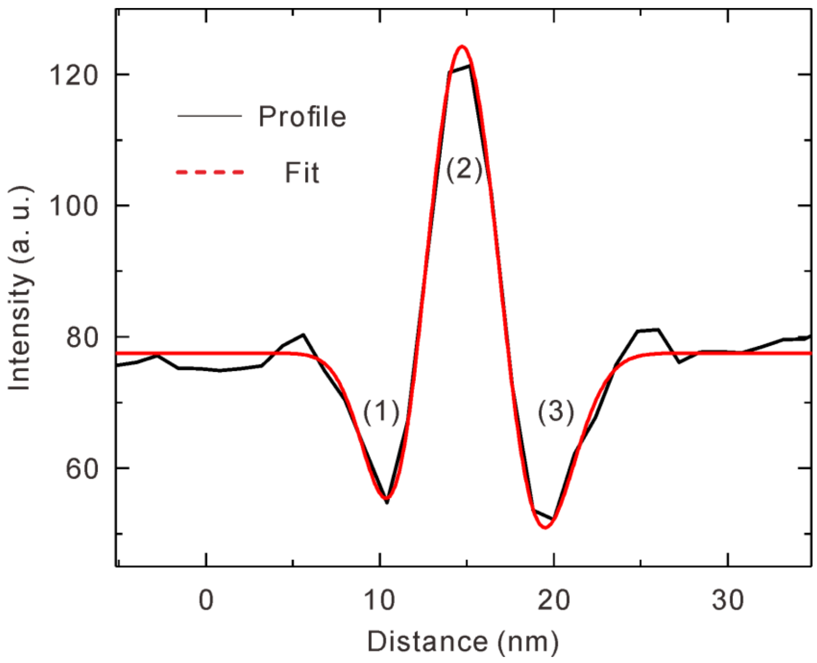

Figure S12. Area-integrated estimation of the amount of deposited (1 and 3) and removed (2) mass. Intensity was fitted with sum of three Gaussian functions. FWHM and absolute peak intensity of each peak are (1) 4.08 nm and 0.135, (2) 5.12 nm and 0.300, and (3) 4.91 nm and 0.178, respectively. The calculated region of each peak was (1) 1.38, (2) 3.85, and (3) 2.19, where sum of two area (1) + (3) = 3.57 was similar to area (2).

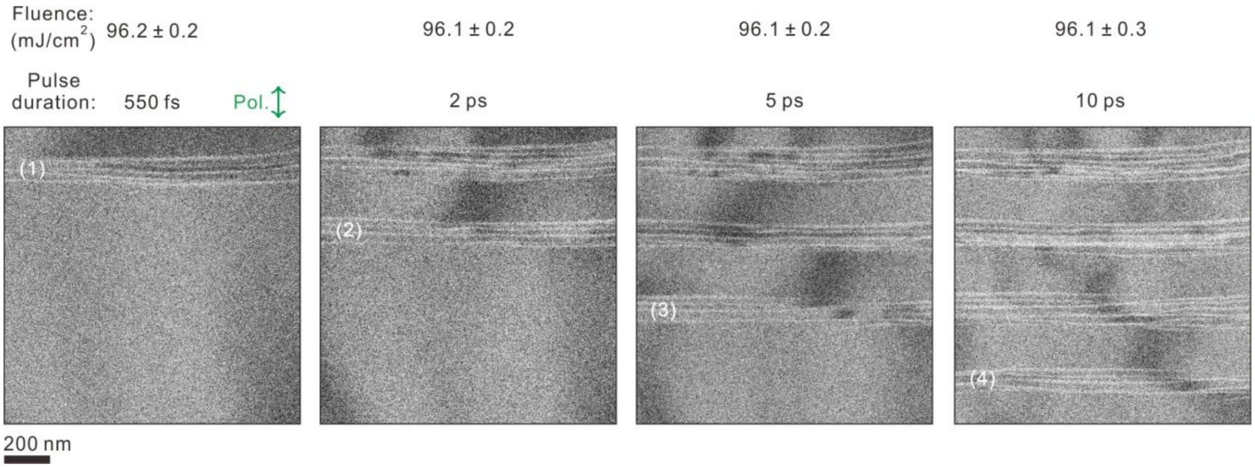

Figure S13. Dependence of the patterning on the laser-pulse duration. Bright-field TEM images of the grooves, which were developed under different durations of laser pulses from 550 fs, 2, 5, and 10 ps from left to right. The test has been made on the same area of the sample (Sample 7) and serially for stringent comparison in an order from 550 fs to 10 ps. Grooves formed with each pulse duration is indicated with arrows. BP was exposed to light for 3−5 s in each case, as short as possible to minimize changes on the preceding grooves but long enough to induce the ablation. Laser fluences for each pulse duration are also denoted. The grooves developed in the same manner regardless of the pulse duration of the incident light up to 10 ps. Sample 7. Scale bar: 200 nm.

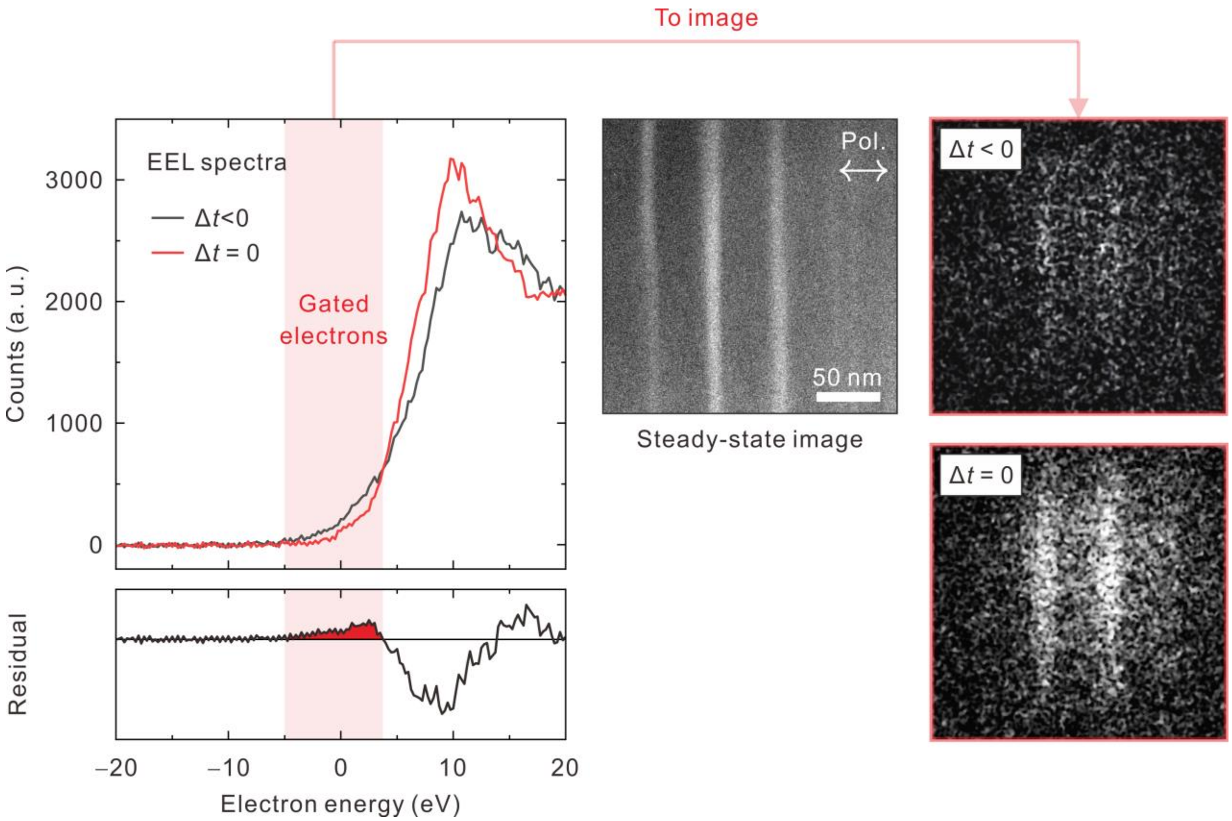

Figure S14. PINEM imaging of field confinement at BP nanogaps. Near-field confinement at the nanogaps was imaged using an energy filter with gated electrons at time zero ($\Delta t = 0$, when the optical pump and electron probe pulses spatiotemporally overlapped at the specimen)[5]. Fluence was set to 30 mJ/cm^2 well below the ablation threshold. The most enhanced near-field was obtained when the polarization of the incident light was parallel to the grating vector of the array of BP nanoribbons. Sample 6.

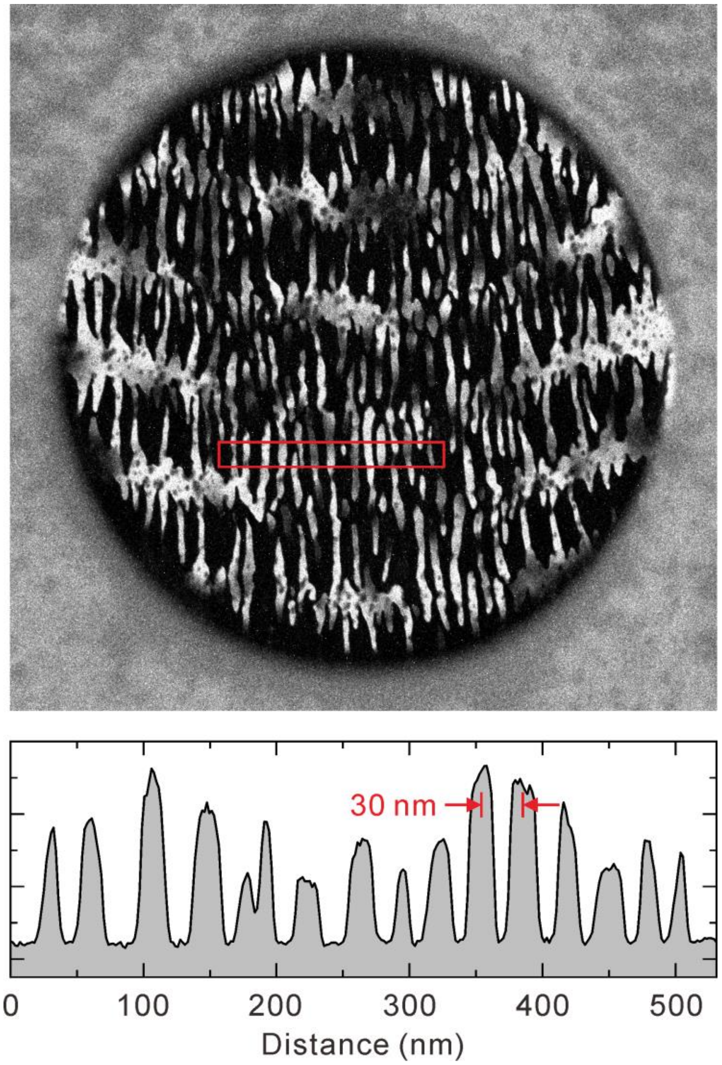

Figure S15. Dark-field TEM image of periodically ablated BP with a 30-nm spacing. The periodic ablation was made on a holey Si_3N_4 substrate upon ultrashort UV (257 nm) pulse irradiation and a larger periodicity (~150 nm) resulting from SW. Dark region is a hole in the substrate. Excitation with large excess energy resulting in nonuniform thermal ablation and lower ablation threshold in BP for UV pulses than 515-nm pulses seem to result in the irregular structures and significant damage. Sample 9.

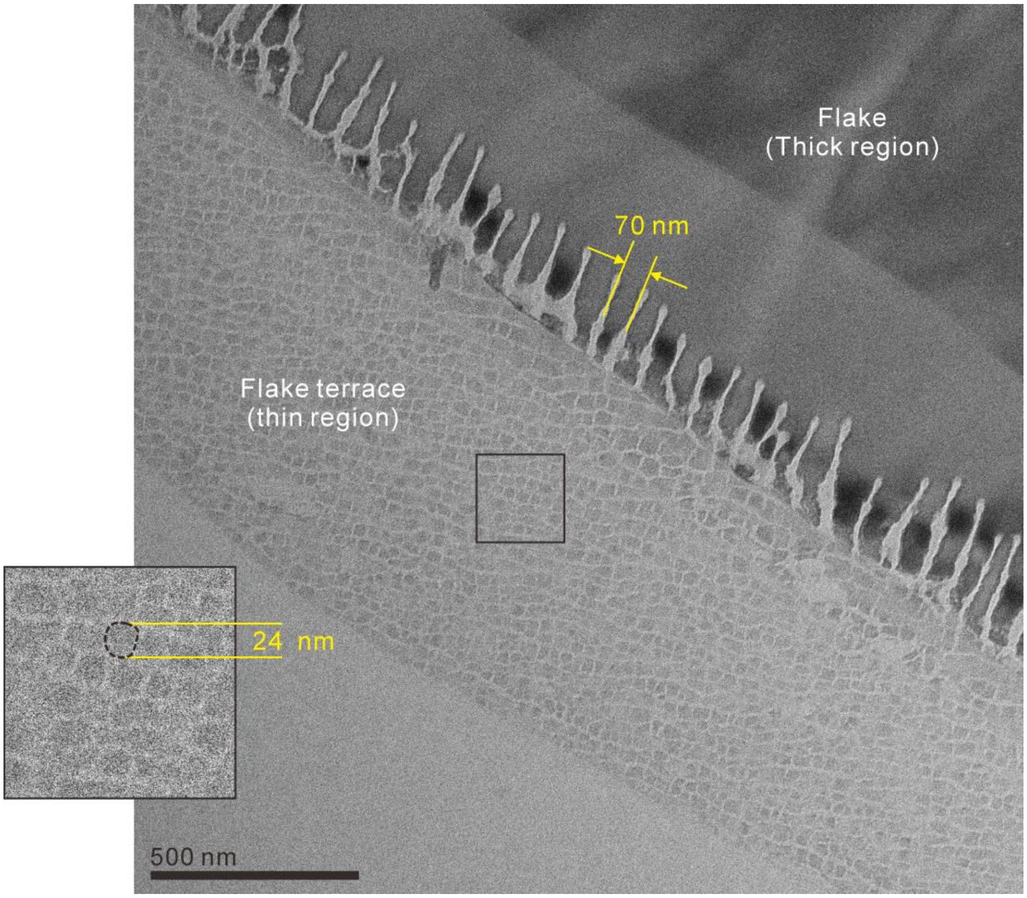

Figure S16. TEM image displaying thickness dependence of tailoring fine-spaced BP nanostructures. The thin terrace (about 10 layers) at boundary of flake (Sample 8) was cut into nanocubes/cuboids (or dots) with a lateral length reaching 24 nm, one-third of the spacing in the 28-layered thicker region under successive irradiation of two perpendicularly polarized light.

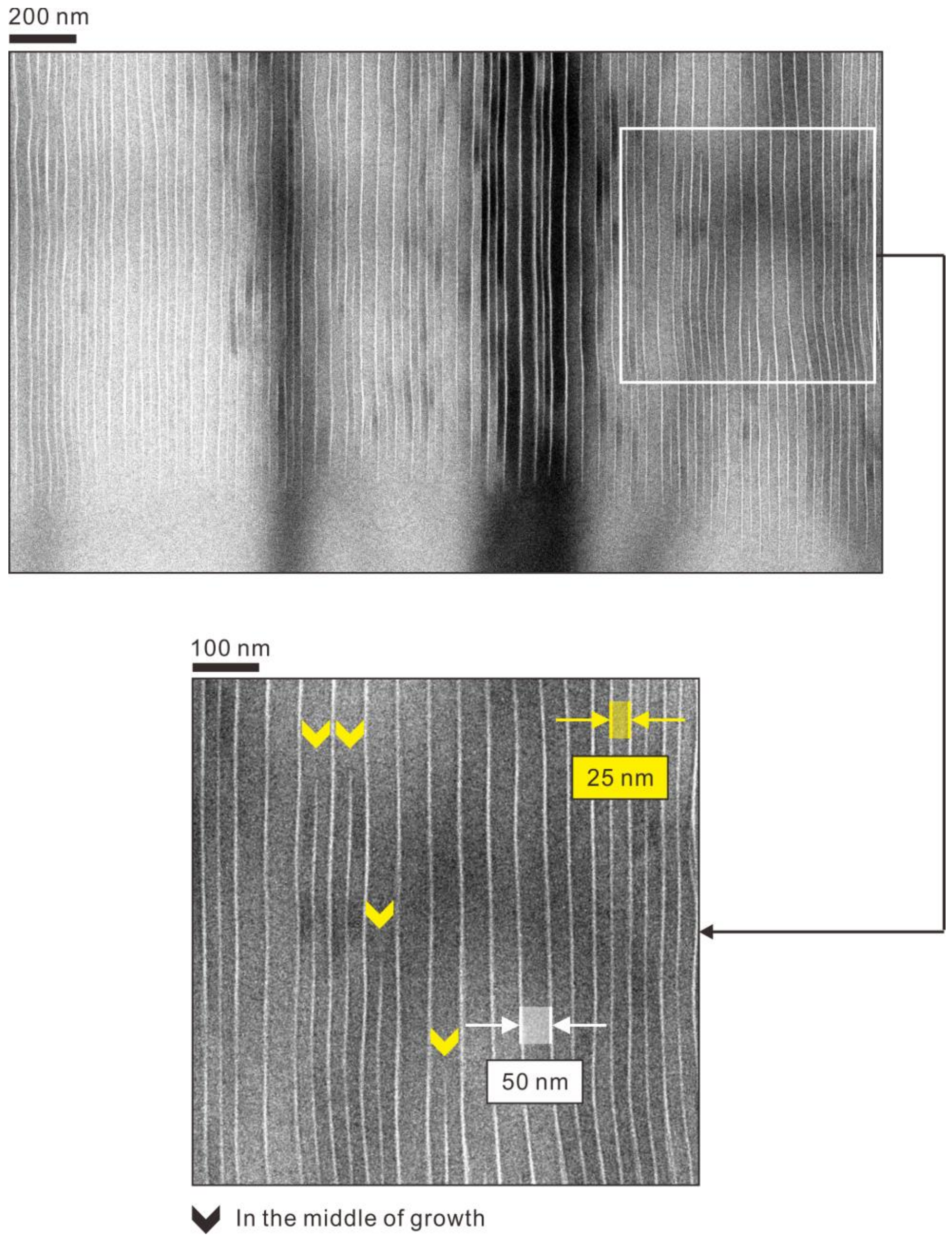

Figure S17. Doubling of spatial frequency. Under the same irradiation condition within the illumination area, the spatial frequency of periodic structures was intermittently doubled to $\lambda_0/20$. This is attributed to the resonance of the MI fields between the initially formed grooves with the $\lambda_0/10$ spacing. The head of the secondary grooves in the middle of growth is indicated with yellow arrows. Constructive resonance of the MI fields confined between the early structures was exploited to create periodic structures with extreme deep-subwavelength spacings.

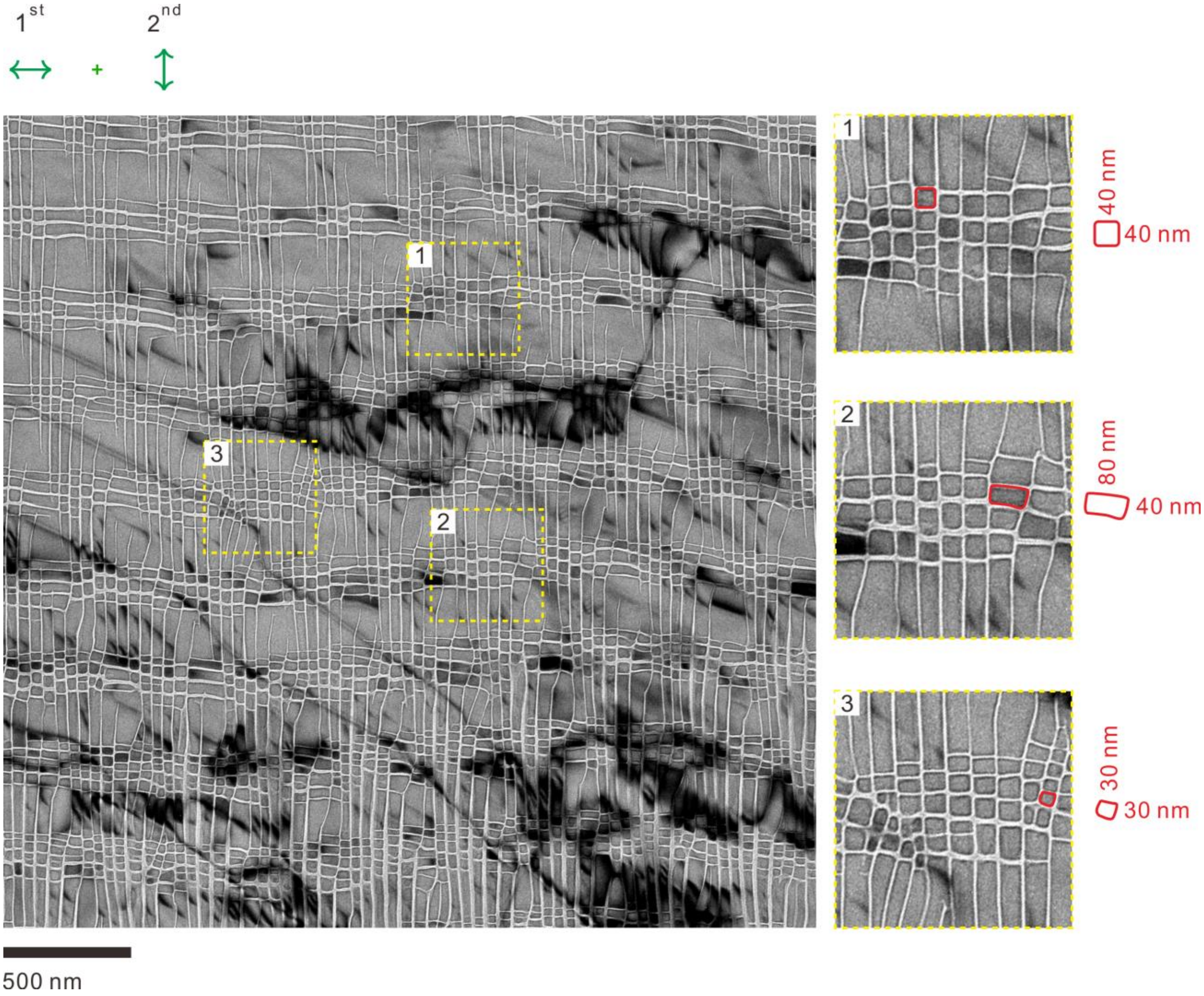

Figure S18. Tailoring nanocubes/cuboids by successive illumination of two orthogonally polarized light (Sample 2). Dimension, shape, and aspect ratio of the nanocubes/cuboids depend on propagation direction of SW, surface roughness, and topography, regardless of the incidence order of light polarization. Nanocubes/cuboids were formed where the two perpendicularly propagating SW waves accompanying the MI fields overlapped. Enlarged views at three different regions of 1–3 are displayed in the right panels. Initial structures upon the first irradiation (N = 1000) were periodic nanoribbons with 50-nm spacing, whereas the secondary ones discontinuously developed along its growth direction.

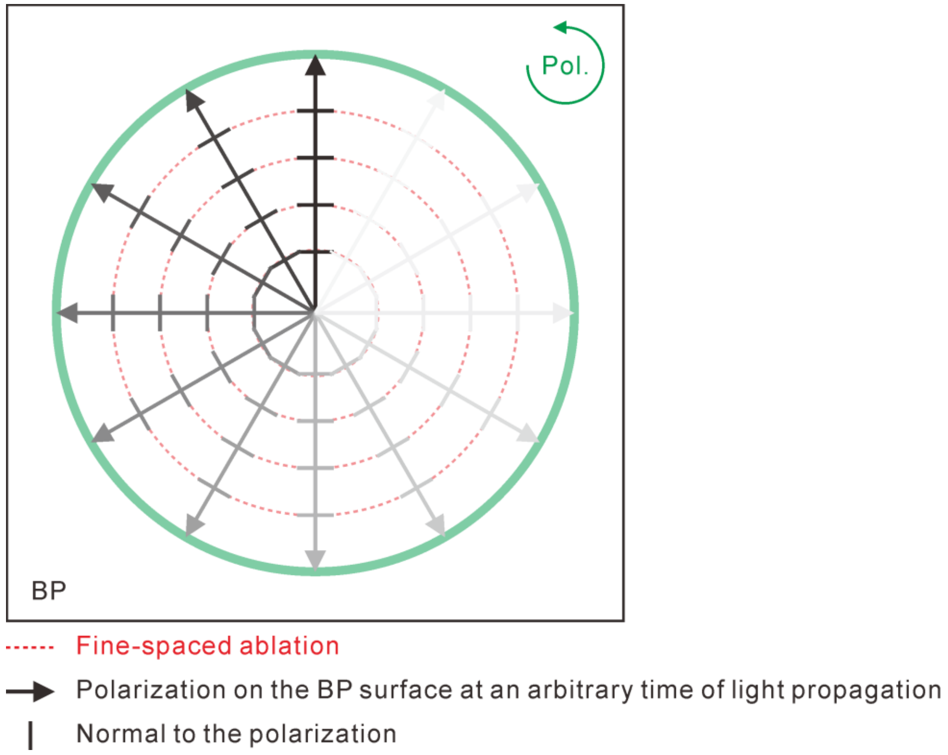

Figure S19. Mechanism of BP nanoring formation with circularly polarized light. In case linearly polarized light was incident on BP, fine-spaced periodic ablation occurred along the normal to the polarization by MI. Similarly, upon applying circularly polarized light, patterns were generated along the normal direction to the rapidly rotating linear polarization and were developed following the handedness of the incident light polarization.

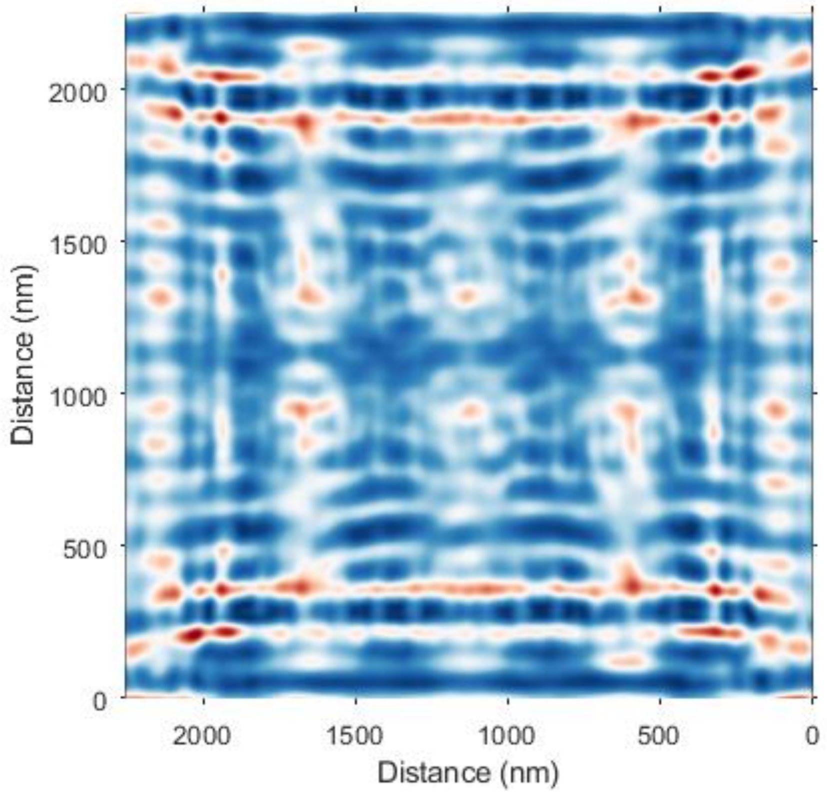

Figure S20. Simulation of a MI electric field upon irradiation with circularly polarized light. 2D electric field intensity profile of rotating MI fields on BP surface under clockwise circularly polarized light is presented. Fluence: 90 mJ/cm^2.

III. Captions for Movies S1 to S4

Movie 1. *In-situ* imaging of BP nanoribbon formation with linearly polarized light. SW is not launched. F = 90 mJ/cm^2.

Movie 2. *In-situ* imaging of BP nanoribbon formation with linearly polarized light. Orientation between the propagation direction of SW and the grating vector of the MI fields is perpendicular. F = 96 mJ/cm^2.

Movie 3. *In-situ* imaging of BP nanocubes/cuboids formation with second linearly polarized light (perpendicular to the first light polarization). SW is not launched. F = 90 mJ/cm^2.

Movie 4. *In-situ* imaging of BP nanoring formation with circularly polarized light (clockwise). SW is launched. F = 90 mJ/cm^2.

IV. References

[1] R. Malendevich, L. Jankovic, G. Stegeman, J. S. Aitchison, Spatial modulation instability in a Kerr slab waveguide. *Opt. Lett.* **2001**, *26*, 1879–1881.

[2] J. Zhang, X. Yu, W. Han, B. Lv, X. Li, S. Xiao, Y. Gao, and J. He, Broadband spatial self-phase modulation of black phosphorous. *Opt. Lett.*, **2016** *41*, 1704–1707.

[3] G. P. Agrawal, *Nonlinear fiber optics* (Academic Press, ed. 5, 2013)

[4] C. Varin, R. Emms, G. Bart, T. Fennel, T. Brabec, Explicit formulation of second and third order optical nonlinearity in the FDTD framework. *Comput. Phys. Commun.* **2018**, *222*, 70–83.

[5] D. J. Flannigan, B. Barwick, A. H. Zewail, Biological imaging with 4D ultrafast electron microscopy. *Proc. Natl. Acad. Sci. U.S.A.* **2010**, *107*, 9933–9937.
